# Competition between *Pseudomonas aeruginosa* and *Staphylococcus aureus* is dependent on intercellular signaling and regulated by the NtrBC two-component system

**DOI:** 10.1038/s41598-022-12650-2

**Published:** 2022-05-30

**Authors:** Morgan A. Alford, Simranpreet Mann, Noushin Akhoundsadegh, Robert E. W. Hancock

**Affiliations:** Centre for Microbial Diseases and Immunity Research and Department of Microbiology, University of British Columbia, Vancouver, BC Canada

**Keywords:** Immunology, Microbiology

## Abstract

*Pseudomonas aeruginosa* and *Staphylococcus aureus* are often comorbid human pathogens, isolated from expectorated sputum of cystic fibrosis patients and chronically infected wounds. Prior studies revealed a competitive advantage of *P. aeruginosa* over *S. aureus* in vitro that was slightly muted in vivo. Here, we demonstrated that the two-component regulatory system NtrBC influences the competitive advantage of *P. aeruginosa* over *S. aureus* in skin organoid and mouse models of co-infection. Expression of *ntrBC* was induced during co-culture of the two species and could be recapitulated in monoculture by the addition of the metabolite N-acetylglucosamine that is released from *S. aureus* following lysis. *P. aeruginosa* LESB58 WT, but not mutant (Δ*ntrC* and Δ*ntrBC*) strains, induced lysis of *S. aureus* USA300 LAC during planktonic growth and outcompeted *S. aureus* USA300 LAC during biofilm formation in vitro. We confirmed these findings in a murine abscess model of high-density infection. Accordingly, the secretory profile of *P. aeruginosa* LESB58 mutants revealed reduced production of anti-staphylococcal virulence factors including pyoverdine, pyocyanin and elastase. These phenotypes of LESB58 Δ*ntrBC* could be at least partly complemented by overexpression of quorum sensing molecules including homoserine lactones or alkylquinolone signaling molecules. These data implicate the NtrBC two-component system in the complex regulatory cascade triggered by interspecies signaling that gives *P. aeruginosa* LESB58 a competitive edge over *S. aureus* USA300 LAC.

## Introduction

*Pseudomonas aeruginosa* is an opportunistic pathogen implicated in infections of different tissues that are increasingly difficult to treat due to the numerous intrinsic, acquired, and adaptive antibiotic resistance mechanisms it employs^1^. *P. aeruginosa* is most commonly co-isolated with *Staphylococcus aureus* from chronic skin wound infections^2,3^ and the expectorated sputum of adults with cystic fibrosis (CF)^4^. Various studies have examined the relationship between *P. aeruginosa* and *S. aureus* in CF lung infection models^5,6^. During early childhood, CF lungs are readily colonized by *S. aureus*, with a higher likelihood of colonization by *P. aeruginosa* in the mid- to late-teenage years^7^. Once present, *P. aeruginosa* rapidly takes over, indicating a potential competitive exclusion of *S. aureus* in the context of CF.

Less is known about the relationship between these species in polymicrobial infections outside the context of CF. However, recent data indicates that the competitive advantage of *P. aeruginosa* over *S. aureus* is muted in the presence of certain host factors^8^. For example, acute wound infection models that incorporate serum into the growth medium allowed *P. aeruginosa* and *S. aureus* to co-exist^2,3^, at least in the early stages of infection. Thus, interspecific interactions appeared to be highly regulated and dependent on environmental conditions. Since intercellular signaling impacts on the production of virulence factors^6,9^, including pyoverdine, pyocyanin and elastases that are excreted from *P. aeruginosa*, interspecies interactions could represent a determinant of virulence.

The *P. aeruginosa* genome is well endowed with regulatory elements, comprising nearly 10% of all genes, including those involved in sensing and rapidly responding to dynamic environmental conditions^10^. Two-component systems, canonically comprising a sensor kinase and a response regulator, are a class of regulatory element that is involved in the rapid adaptation to environmental conditions through signal transduction leading to the expression of effectors important for adaptation^11^. The NtrBC two-component system, comprised of the sensor kinase NtrB and the response regulator NtrC, is important for regulating nitrogen metabolism during nutrient limitation^12^. Some response regulators such as NtrC belong to a subclass known as bacterial enhancer binding proteins^12^ that promote transcription of genes from RpoN (σ^54^)-dependent promoters, though they may also regulate gene expression independent of σ^54^^13^. Genes in the NtrC regulon^14,15^ are involved in surface colonization (e.g., *muc* operon), virulence in acute and chronic infections (e.g., *algU*, *pvdD*, *pscH*, *phuR*) and scavenging of nutrients (e.g., *nap*, *nas*, *nir* operons), some of which have no annotated σ^54^ binding site. Accordingly, we previously demonstrated^15,16^ that NtrBC regulated several adaptive lifestyles of *P. aeruginosa* including biofilm formation in vitro, colonization in a subcutaneous infection model in vivo and expression of virulence factors.

Here, we aimed to elucidate the role of NtrBC signaling in interspecies competition between *P. aeruginosa* and *S. aureus* since expression of NtrC had been shown to be induced in the early stages of co-culture^17^. It was confirmed that *ntrBC* promoter activity of the *P. aeruginosa* Liverpool Epidemic Strain (LES)B58 was induced in the presence of the community-acquired methicillin resistant *S. aureus* (MRSA) clinical isolate USA300 LAC, as well as the small molecule N-acetylglucosamine that is liberated from *S. aureus*. *P. aeruginosa* LESB58 wild-type (WT) and an isogenic Δ*ntrB* mutant outgrew and induced lysis of *S. aureus* USA300 LAC in competition assays in vitro, but LESB58 Δ*ntrC* and Δ*ntrBC* strains did not. The staphylolytic activity of LESB58 Δ*ntrC* and Δ*ntrBC* strains could be complemented by overexpression of genes encoding quorum sensing (QS) signaling molecules including *lasI* and *pqsH* but not *rhlI*, at least in part by restoring the production of *Pseudomonas* anti-staphylococcal virulence factors. Importantly, the *ntrBC*-dependent competitive phenotypes were maintained, albeit somewhat muted, during biofilm formation in more complex human and mouse models of co-infection. Based on these data, we propose a model by which NtrBC activity could shape interspecies interactions between *P. aeruginosa* and *S. aureus* during the early stages of co-culture.

## Results

**Figure 1:**
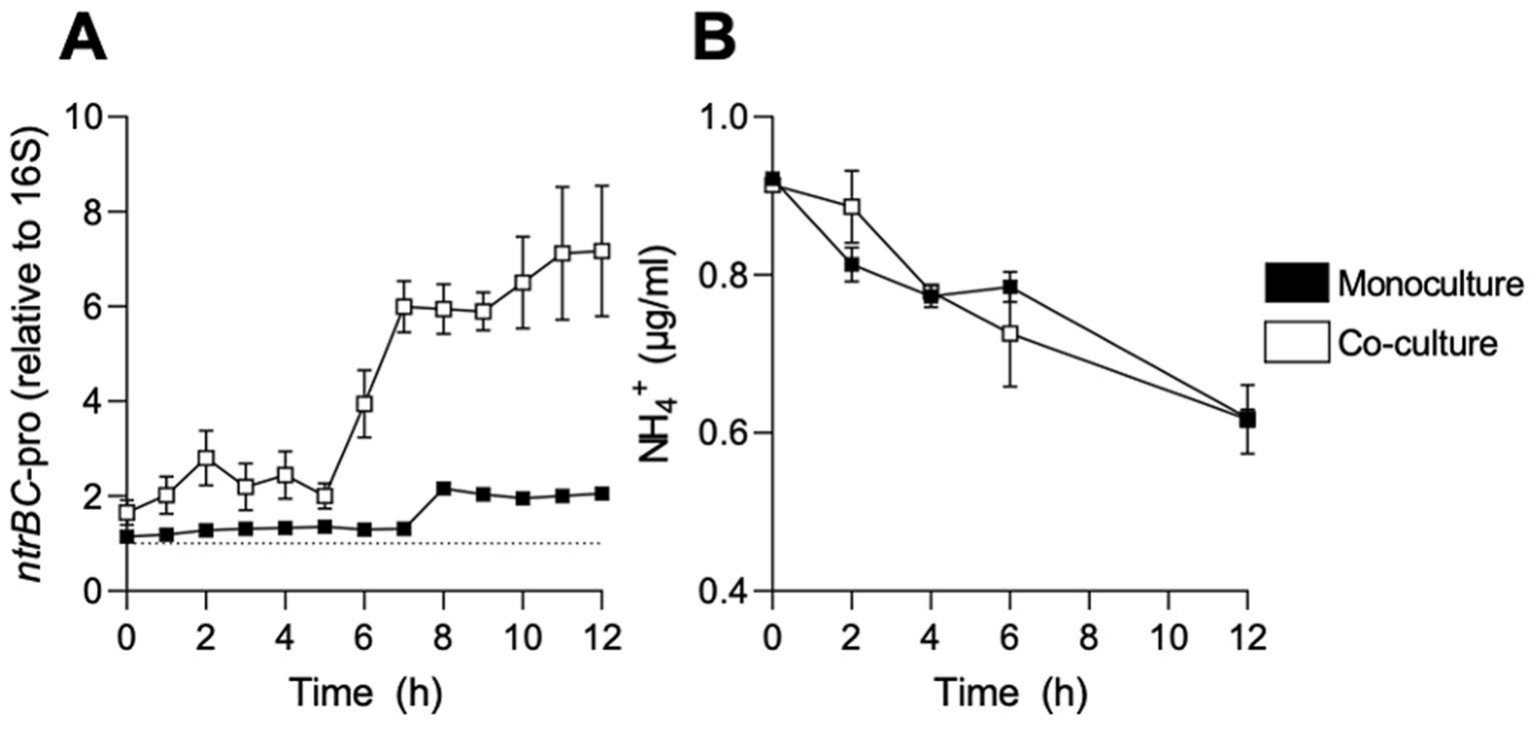
Induction of *P. aeruginosa* LESB58 *ntrBC* promoter activity (*ntrBC*-pro) during co-culture with *S. aureus*. (**A**) *P. aeruginosa* LESB58 was seeded in the absence (shown as monoculture in black) or presence (shown as co-culture in white) of *S. aureus* USA300 LAC at a total bacterial density of ~ 5 × 10^5^ CFU/ml and luminescence due to the activation of the *ntrBC* promoter was monitored every h for up to 12 h. (**B**) Lack of dependence on ammonium depletion from the medium. Extracellular concentration of ammonium (NH_4_ +) was monitored in parallel using an ammonia assay kit. Data are presented as mean ± standard error of the mean (SEM) for three independent experiments containing three technical replicates in each (*n* = 3).

To confirm the finding that *P. aeruginosa ntrBC* expression was stimulated in the early stages of co-culture with *S. aureus*^17^, LESB58 *ntrBC* promoter activity was monitored by luminescence detection in the presence or absence of USA300 LAC (Fig. 1A). Ammonium concentration in the medium was measured in parallel (Fig. 1B), since depletion of extracellular ammonium was correlated with low intracellular nitrogen availability and NtrC activation of other reference strains of *P. aeruginosa*^18,19^.

The luminescence (i.e., *ntrBC* promoter activity) detected from co-culture increased rapidly six h after inoculation. At six and seven h post-inoculation, *ntrBC* promoter activity in co-culture was 2.6- and 4.7-fold greater, respectively, than the promoter activity in monoculture (Fig. 1A). The maximum luminescence detected during co-culture was 5.1-fold greater than during monoculture at 12 h post-inoculation (7.2 versus 2.1). Ammonium depleted slowly and at similar rates during both mono- and co-culture of species (Fig. 1B), indicating that LESB58 *ntrBC* promoter activity was independent of extracellular ammonium levels. Indeed, ammonium was only reduced by 32.6% during monoculture, from 0.92 μg/ml at the time of inoculation to 0.62 μg/ml 12 h post-inoculation. Similarly, ammonium was reduced by 31.9% during co-culture, from 0.91 μg/ml at the time of inoculation to 0.62 μg/ml 12 h post-inoculation.

**Figure 2:**
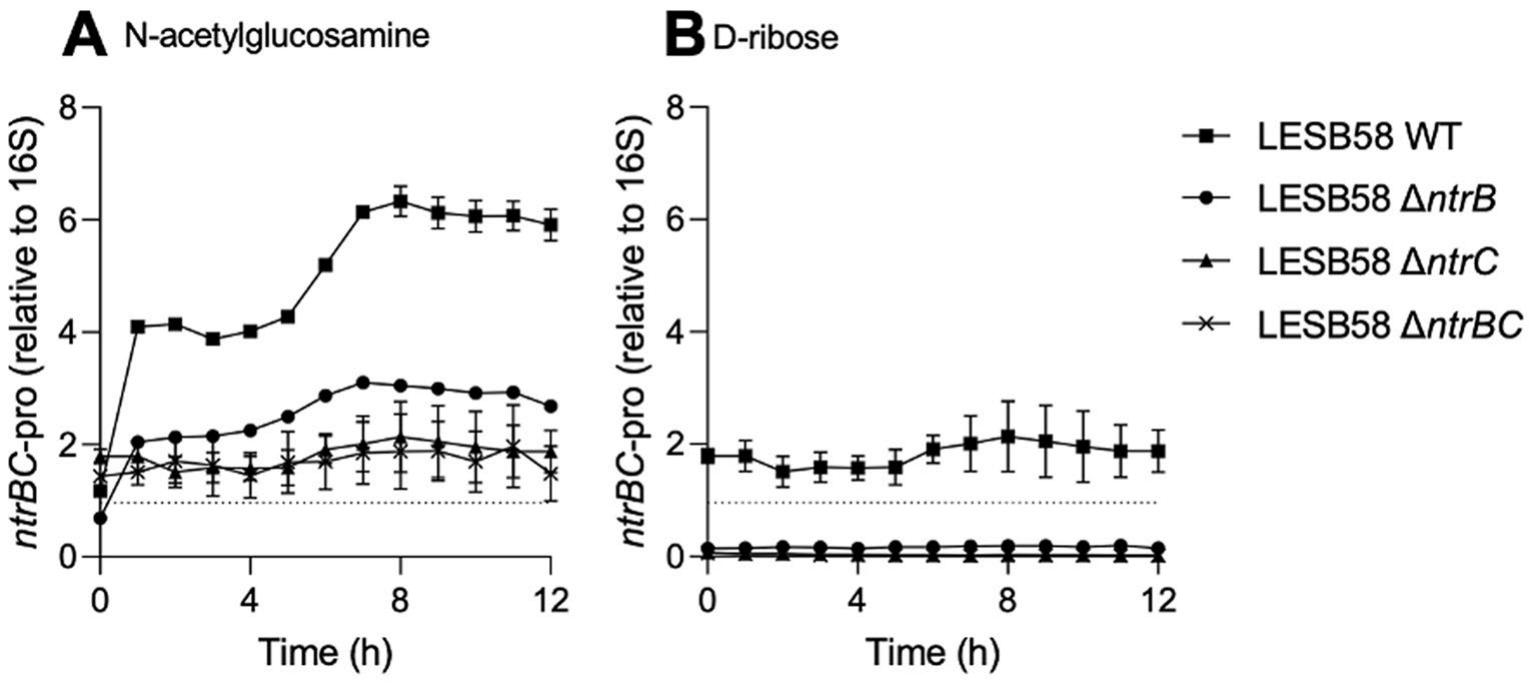
Induction in *P. aeruginosa ntrBC* promoter activity by *S. aureus* small molecules**.** N-acetylglucosamine and to a lesser extent D-ribose caused the induction of *P. aeruginosa* LESB58 *ntrBC* promoter activity (*ntrBC*-pro). *P. aeruginosa* LESB58 strains were seeded at a density of ~ 5 × 10^5^ CFU/ml and treated with (**A**) N-acetylglucosamine (20 μM) or (**B**) D-ribose (20 μM) prior to luminescence detection for up to 12 h. Data are presented as mean ± standard error of the mean (SEM) for three independent experiments containing three technical replicates in each (*n* = 3).

N-acetylglucosamine is a component of peptidoglycan that can be liberated following bacterial (e.g., *S. aureus*) lysis, and D-ribose is an analogue of the autoinducer-2 QS molecule produced by Gram-positive pathogens (e.g., *S. aureus*)^20^. To determine whether these signaling molecules had a potential role in inducing *ntrBC* promoter activity during co-culture, we examined the impact of N-acetylglucosamine and D-ribose on luminescence of LESB58 WT, Δ*ntrB*, Δ*ntrC* and Δ*ntrBC* mutants (Fig. 2).

N-acetylglucosamine treatment stimulated *ntrBC* promoter activity of LESB58 WT by 2.9-fold one h post-inoculation, relative to *t* = 0 h, rising to 5.2-fold eight h post-inoculation. The promoter activity of *ntrBC* began to decline thereafter, although was still 4.7-fold greater 12 h post-inoculation (Fig. 2A). Only a 0.35- to 0.45-fold increase in promoter activity was observed in Δ*ntrC* or Δ*ntrBC* mutants at the peak level at *t* = 8 h, although they always exhibited *ntrBC* promoter activity that was greater than 16S promoter activity. In contrast, the Δ*ntrB* mutant had an activity that was intermediate between these mutants and WT, demonstrating a 2.4-fold induction of *ntrBC* promoter activity in the presence of N-acetylglucosamine eight h post-inoculation. D-ribose induced the expression of *ntrBC* in the LESB58 WT only, which exhibited a 0.34-fold increase in luminescence at *t* = 8 h than *t* = 0 h (Fig. 2B), although *ntrBC* promoter activity was always greater than 16S promoter activity under these conditions. In the mutants there was no induction of *ntrBC* promoter activity, indicating that the effect of ribose was dependent on NtrBC. These data were consistent with at least a partial requirement of NtrB and NtrC for the induction of *ntrBC* promoter activity by *S. aureus* metabolites.

**Figure 3:**
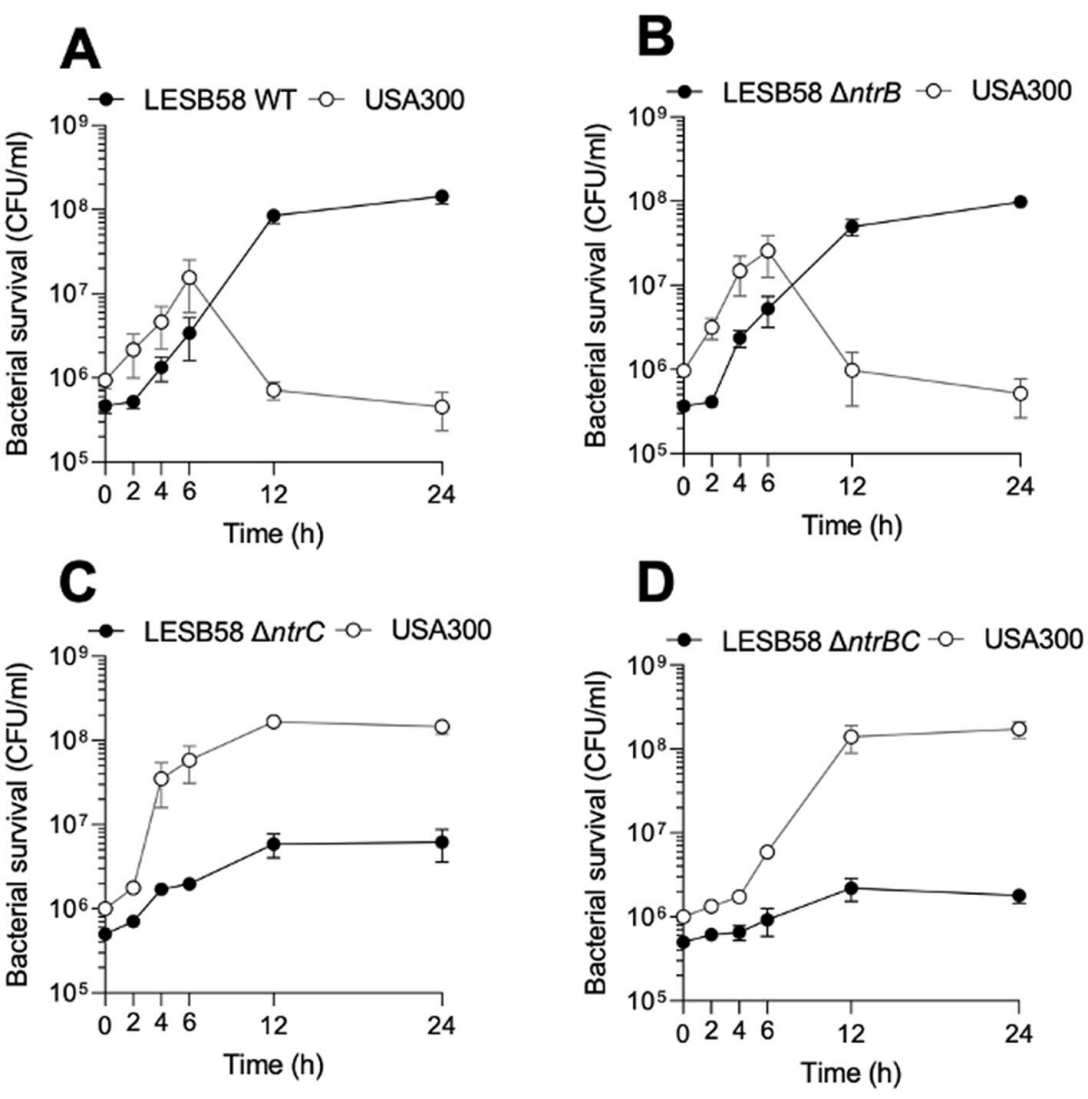
*P. aeruginosa* LESB58 outcompeted *S. aureus* USA300 LAC in an NtrC-dependent manner. *P. aeruginosa* LESB58 (**A**) WT, (**B**) Δ*ntrB*, (**C**) Δ*ntrC* or (**D**) Δ*ntrBC* mutant strains were seeded at a starting OD_600_ = 0.1 in batch cultures that were sampled at two, six or 12 h intervals and plated on selective media for bacterial enumeration. Data are presented as mean ± standard error of the mean (SEM) for three independent experiments (*n* = 3).

Next, we confirmed the observation^20^ that *P. aeruginosa* competitively displaces *S. aureus* using clinical isolates in batch culture growth experiments (Fig. 3A). Since NtrBC promoter activity was stimulated during co-culture with *S. aureus* USA300 LAC, it was hypothesized that NtrB and/or NtrC activity was important for the competitive advantage of *P. aeruginosa* over *S. aureus*. It was observed that *P. aeruginosa* LESB58 Δ*ntrC* and Δ*ntrBC* mutants were outcompeted by *S. aureus* USA300 LAC during batch culture in BM2 (Fig. 3C,D), whereas the WT and Δ*ntrB* mutant maintained a competitive edge during co-culture (Fig. 3A,B).

During co-culture with either *P. aeruginosa* LESB58 WT or Δ*ntrB* strains, *S. aureus* USA300 LAC grew steadily until six h post-inoculation (Fig. 3A,B). However, between six and 12 h, death of USA300 LAC was observed, since the number of USA300 LAC recovered from co-culture with LESB58 WT and Δ*ntrB* at the 12 h time point was 21.7-and 26.0-fold less than at six h, respectively. During this period, the growth rate of LESB58 WT was exponential and constant, although LESB58 Δ*ntrB* had a slight reduction in growth rate. This was reflected by the growth constants (*μ*) (Table S3) for LESB58 WT and Δ*ntrB*, which were 0.43/h and 0.40/h during exponential growth, respectively. In contrast, the growth rate of the Δ*ntrC* and Δ*ntrBC* mutants (0.21/h and 0.12/h, respectively) was greatly reduced after the two h time point when compared to LESB58 WT or Δ*ntrB* strains. Furthermore, LESB58 Δ*ntrC* and Δ*ntrBC* mutants never overtook USA300 LAC. *S. aureus* USA300 LAC grew to a total density of 1.4–1.6 × 10^8^ CFU/ml by 12 h post inoculum during co-culture with LESB58 Δ*ntrC* and Δ*ntrBC* mutants, respectively, whereas their density was reduced to 7.2 or 9.8 × 10^5^ CFU/ml during co-culture with WT and Δ*ntrB*, respectively*.* Differences between the strains were ameliorated by complementation of the deleted gene (Fig. S1). This showed that interspecies inhibition of *S. aureus* USA300 LAC by *P. aeruginosa* LESB58 was dependent on NtrBC.

**Figure 4:**
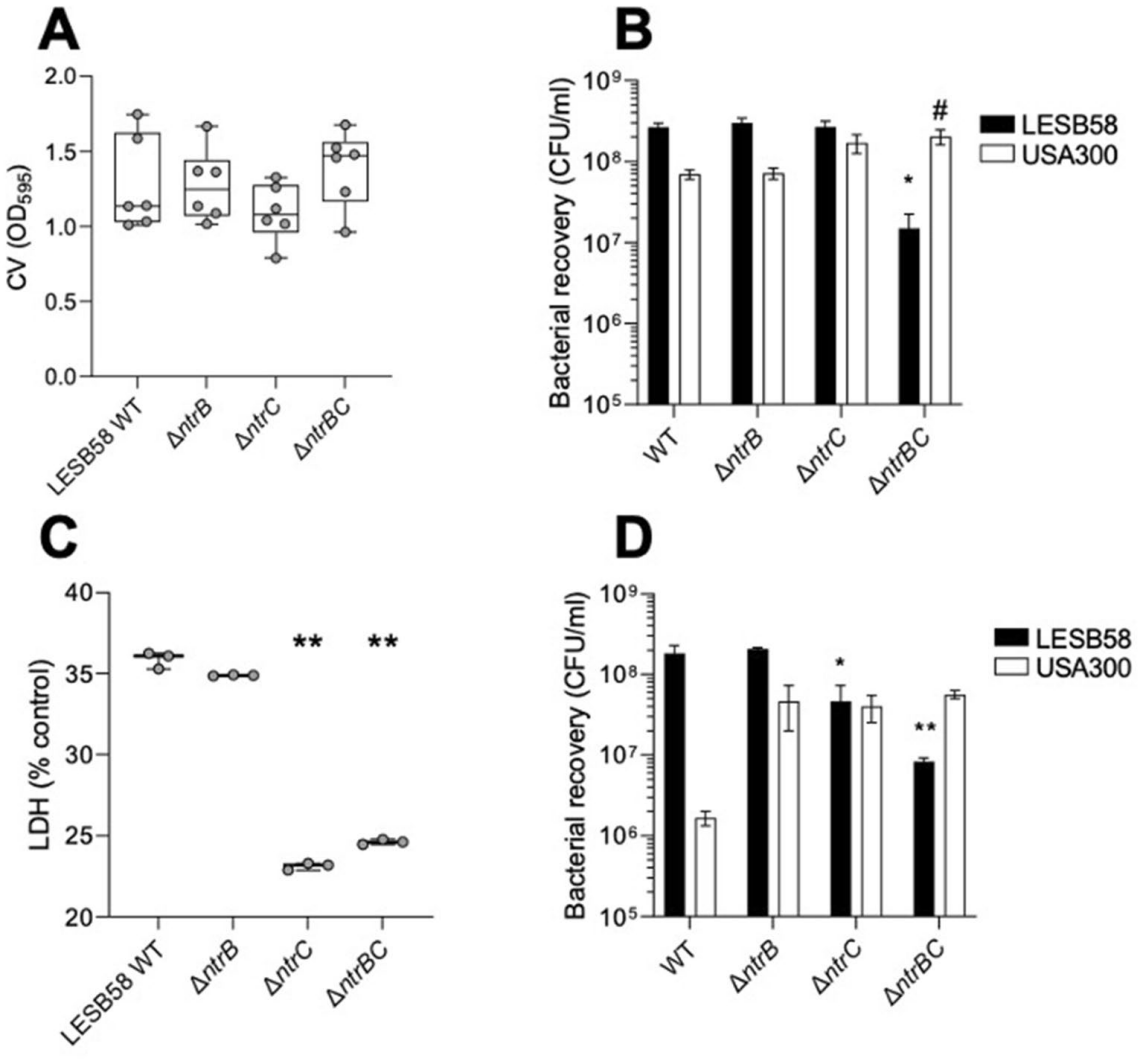
NtrBC-dependent competitive advantage of *P. aeruginosa* LESB58 over *S. aureus* USA300 LAC was observed in human organoids. Biofilms were formed with *P. aeruginosa* LESB58 strains (**A,B**) in DMEM with FBS and glucose on polypropylene plates or (**C,D**) on human skin organoids. In vitro biofilms were **(A)** stained with CV and (**B**) scraped for bacterial enumeration on selective media. Skin organoids (**C**) were assessed for LDH release from skin cells and (**D**) biofilm bacteria were enumerated after 18–24 h. Data are presented as mean ± standard error of the mean (SEM) and box plots delineate interquartile range for data from three independent experiments with one or two biological replicates in each (*n* = 3–6). * *P* < 0.05, ** *P* < 0.01 different than biofilms formed with LESB58 WT according to one-way (**C**) or two-way (**B**,**D**) ANOVA followed by Dunnett’s post-hoc analysis. # *P* < 0.05 different than biofilms formed by USA300 LAC with LESB58 WT according to two-way ANOVA (**B**) followed by Dunnett’s post-hoc analysis.

Next, we determined whether the competitive advantage conferred on *P. aeruginosa* LESB58 by NtrBC depended on environmental conditions since, for example, it had been previously observed that interspecies competition was muted in the presence of host factors^20^. Thus, interspecies competition was examined between LESB58 WT and mutant strains co-cultured with USA300 LAC in biofilm formation assays in vitro (Fig. 4A,B) and in a model of biofilm infection formed on a human skin organoid model (Fig. 4C,D).

The total amount of biomass that was formed by mixed species biofilms in polypropylene 96-well plates containing DMEM supplemented with FBS and glucose was not significantly different between strains (Fig. 4A), although the number (CFU/ml) of LESB58 or USA300 recovered from biofilms varied depending on which strain of LESB58 was co-inoculated (Fig. 4B). In the WT mixed species biofilms, there was much lower competition between *P. aeruginosa* LESB58 and *S. aureus* USA300 with only a 3.6-fold advantage for *P. aeruginosa* (Fig. 4), cf. the > 100-fold difference in broth co-culture (Fig. 3). More specifically, the number of LESB58 Δ*ntrBC* was significantly reduced by 218-fold from 2.4 × 10^8^ CFU/ml to 1.1 × 10^7^ CFU/ml, on average. Accordingly, the number of USA300 LAC increased threefold from 6.2 × 10^7^ CFU/ml (recovered from biofilms formed with LESB58 WT) to 1.9 × 10^8^ CFU/ml (recovered from biofilms formed with LESB58 Δ*ntrBC*). The *P. aeruginosa* LESB58 mutants might have exhibited different competition toward *S. aureus* USA300 LAC in biofilm or planktonic growth assays due to the reduced ability of Δ*ntrBC* to form biofilms even in the absence of competition^15^ or due to the different composition of the medium that might impact on LESB58 Δ*ntrBC* fitness.

Compared to biofilms formed on skin organoids with either USA300 LAC and LESB58 WT, mixed biofilms formed by USA300 LAC and either LESB58 Δ*ntrC* or Δ*ntrBC* caused 12.8% (23.1% cf. 35.9% relative to control) and 11.3% (24.6% cf. 35.9% relative to control) less cytotoxicity in a human skin organoid model (Fig. 4C). In contrast, mixed biofilms formed with USA300 LAC and LESB58 Δ*ntrB* were comparable to that of WT (34.9% cf. 35.9% relative to control). *P. aeruginosa* LESB58 WT was recovered in 100-fold larger numbers than *S. aureus* USA300 LAC (Fig. 4D), similar to the observations in broth co-culture (Fig. 3). Recovery of LESB58 Δ*ntrC* was significantly decreased by 2.8-fold from 1.9 × 10^8^ CFU/ml to 6.9 × 10^7^ CFU/ml, whereas recovery of LESB58 Δ*ntrBC* was reduced even more by 232-fold to 8.2 × 10^6^ CFU/ml (Fig. 4D). In co-culture with all mutant strains, the number of *S. aureus* USA300 LAC was significantly increased by nearly 800-fold from 1.1 × 10^6^ CFU/ml to approximately 8 × 10^8^ CFU/ml (Fig. 4D). Thus, while in vitro biofilms showed somewhat different interspecies competition effects than those observed in batch culture, biofilms on skin organoids showed rather similar effects with modest differences.

**Figure 5:**
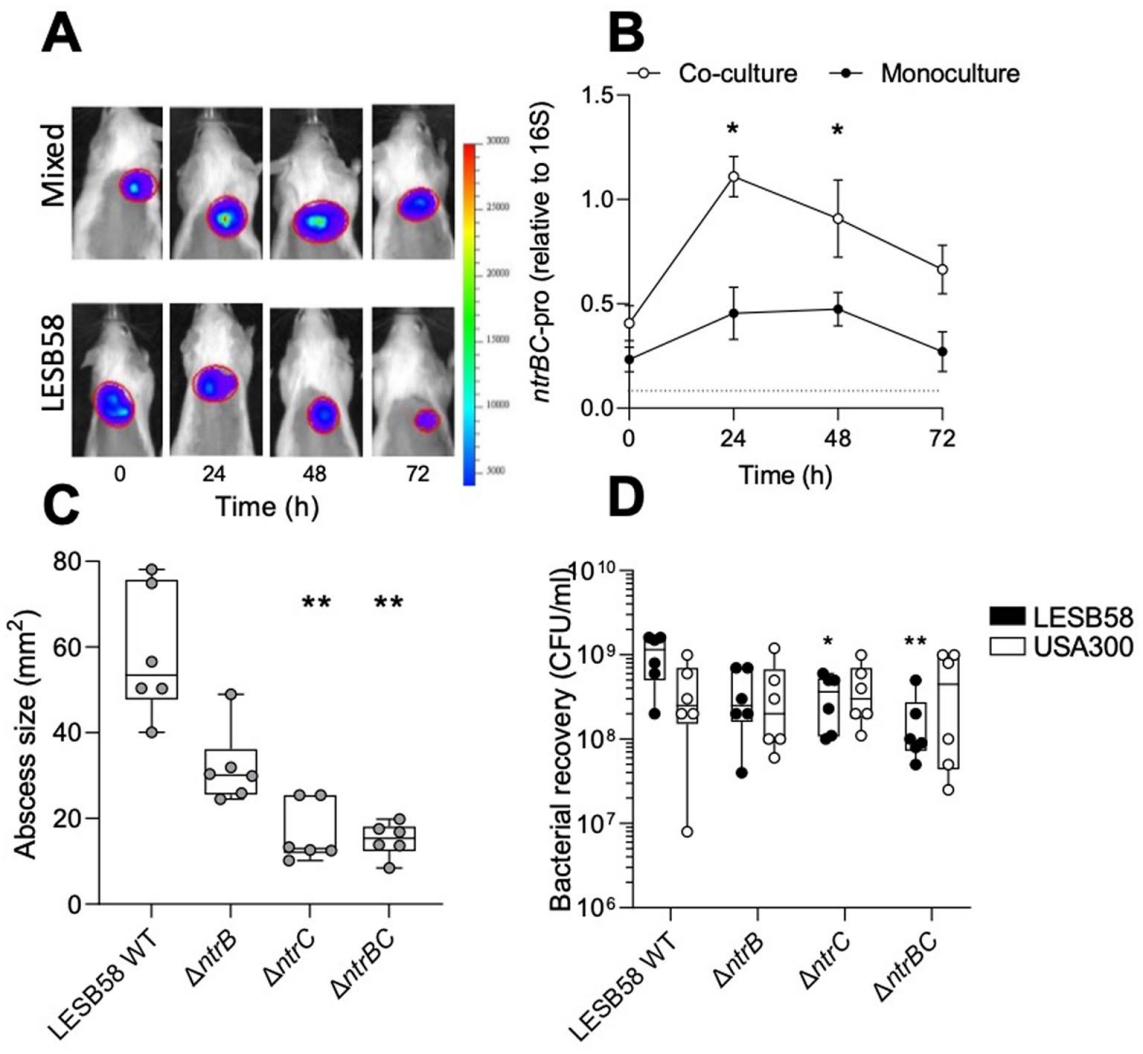
*P. aeruginosa* LESB58 *ntrBC* promoter activity was induced during co-culture in vivo and Δ*ntrC* and Δ*ntrBC* mutants were modestly outcompeted by *S. aureus* USA300 LAC. Bacteria were inoculated in the right dorsum of mice and (**A,B**) luminescence due to the expression of the *lux*-fused *ntrBC* promoter was (**A**) imaged with an in vivo imaging system (IVIS) and (**B**) quantified relative to 16S rRNA promoter activity. After euthanasia (**C**) abscess size was measured using a calliper and (D) live bacteria were enumerated following homogenization, plating on selective media, and overnight growth at 37ºC. Data are presented as mean ± standard error of the mean (SEM), and box plots delineate the interquartile range for data from three independent experiments containing two biological replicates in each (*n* = 6). (**B**) * *P* < 0.05, ** *P* < 0.01 different from mono-species infection according to paired t-test. (**C**) ** *P* < 0.01 different from mixed abscesses formed with LESB58 WT according to Kruskal–Wallis test followed by Dunn’s correction. (**D**) * *P* < 0.05, ** *P* < 0.01 different from LESB58 WT recovered from mixed abscesses according to two-way Kruskal Wallis test followed by Dunn’s correction.

To determine the importance of NtrBC on competition in vivo, the murine abscess model of high-density infection^21^ was modified by co-inoculating LESB58 strains and USA300 LAC (Fig. 5). The induction of *ntrBC* promoter activity was first assessed in vivo (Fig. 5A,B). Relative to 16S rRNA expression, there was a 2.9-fold greater *ntrBC* promoter activity observed during polymicrobial infection than monomicrobial infection at 24 h post-infection. Thereafter, the *ntrBC* promoter activity observed during polymicrobial infection declined, but was still higher than the activity observed during mono-species infection, although promoter activity during multi-species infection was only significantly greater than mono-species infection at the 48 h time-point (Fig. 5B).

The area of abscesses formed with USA300 LAC mixed with LESB58 WT were, on average, 58.3 mm^2^, whereas polymicrobial abscesses formed with the mutants were only 31.1, 19.2 or 19.3 mm^2^ (Fig. 5C), but were only statistically significant for Δ*ntrC* and Δ*ntrBC*. There were no statistically significant differences between the numbers of bacteria recovered from polymicrobial abscesses formed with LESB58 WT and Δ*ntrB* (Fig. 5D). However, in mixed infections the average numbers of LESB58 Δ*ntrC* were reduced threefold from 1.0 × 10^9^ CFU/ml to 3.4 × 10^8^ CFU/ml, and 5.3-fold for LESB58 Δ*ntrBC,* to 1.9 × 10^8^ CFU/ml (Fig. 5D).

To begin to unravel the mechanism(s) by which NtrBC conferred a competitive advantage on *P. aeruginosa* LESB58 over *S. aureus* USA300 LAC, the regions upstream of all coding sequences in *P. aeruginosa* were scanned for the NtrC binding motif^22^ (Fig. S2) using FIMO software^23^. FIMO detected 259 binding targets (Table S4), some having more than one non-redundant binding site; 36 of the downstream genes were differentially expressed in PA14 Δ*ntrB* or Δ*ntrC* strains^15^. A literature search identified strong possibilities from initial hits that were involved in the production of anti-Staphylococcal virulence factors and were expressed from RpoN-dependent promoters, and differential expression of these genes was confirmed using RT-qPCR (Table 1).

**Table 1:** Differential expression of selected genes involved in the production of anti-Staphylococcal virulence factors.

LESB58 Locus Tag	Gene Name	Annotation	Fold Change cf. WT		
			Δ*ntrB*	Δ*ntrC*	Δ*ntrBC*
PALES_07171	*phzA1*	Phenazine biosynthesis protein	− 10.1	− 9.8	− 9.2
PALES_41691	*pys2*	Pyocin S2	− 6.6	− 6.3	− 6.8
PALES_45811	*algU*	RNA Polymerase sigma factor AlgU	− 5.4	− 5.6	− 5.4
PALES_28691	*pvdS*	Extracytoplasmic function sigma factor	− 4.5	− 4.1	− 4.3
PALES_39841	*lasR*	Transcriptional regulator LasR	− 3.9	− 4.1	− 4.0
PALES_44741	*plcH*	Hemolytic phospholipase C precursor	2.1	2.0	2.4

Gene expression is shown as fold-change (FC) from WT using the ΔΔ*Ct* method. Dysregulated expression was considered meaningful when FC was greater than ± 2. Data are shown as the mean from three independent experiments each containing three technical replicates.

According to RT-qPCR, the most significantly downregulated hit was *phzA1*, a phenazine biosynthesis protein^24^ that was 9.2- to 10.1-fold downregulated when compared to WT. Since *phzA2* exhibited high percent identity (97.5%) with *phzA1*, it is possible that this result stemmed from dysregulation of either or both genes. The next most downregulated hit was *pys2*, a pyocin with antibacterial impacts on competitors^25^, which was 6.3- to 6.8-fold downregulated. Other downregulated genes included transcriptional regulators such as *algU*, *pvdS* and *lasR*, the last of which is a master regulator of QS in the hierarchical regulatory network of *P. aeruginosa*^26^ and has impacts on production of virulence factors with anti-Staphylococcal activity. In contrast, phospholipase C was repressed by *ntrBC*.

**Figure 6:**
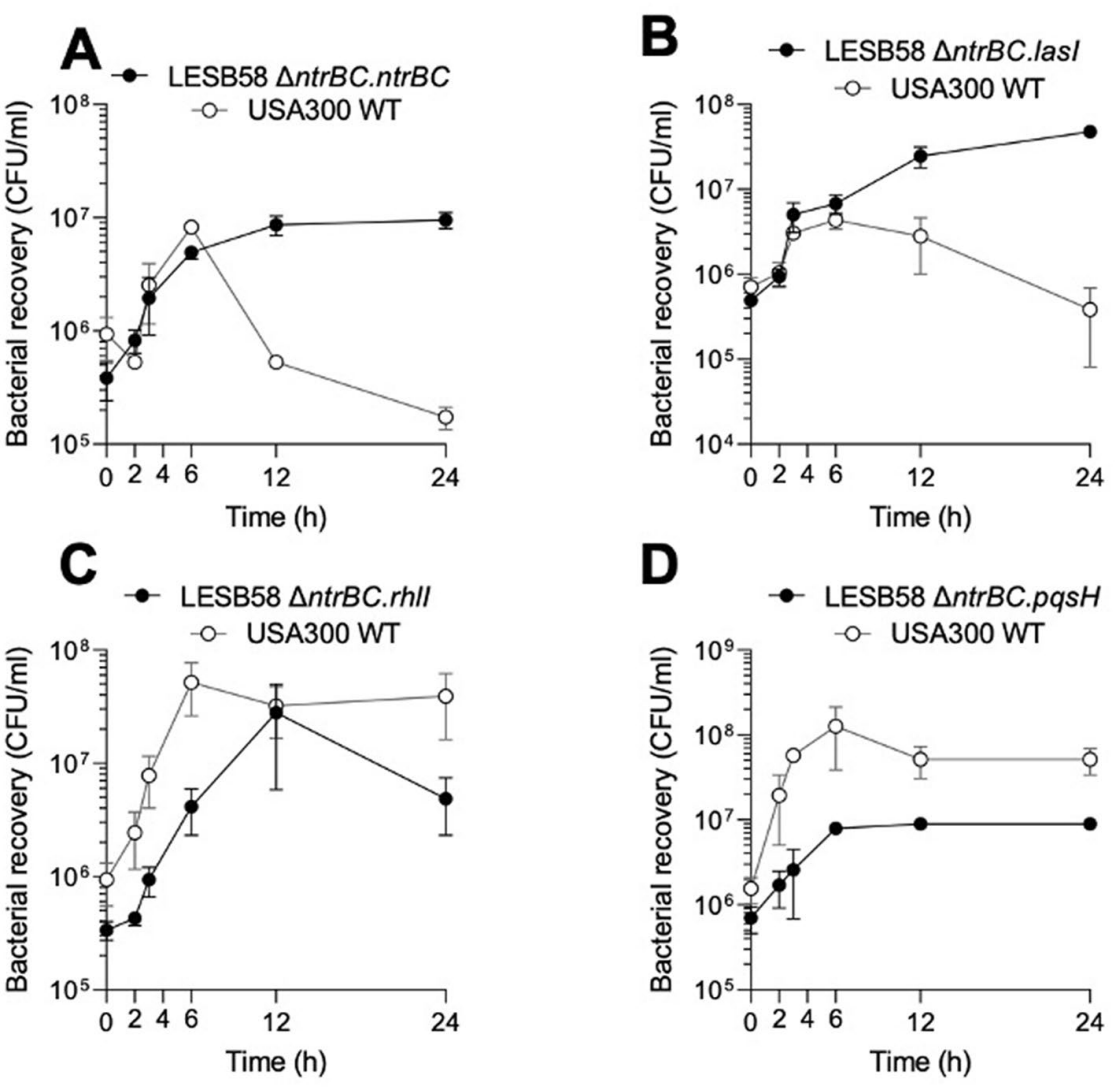
Competitive phenotype of *P. aeruginosa* LESB58 Δ*ntrBC* could be phenotypically complemented. Phenotypic complementation by (**A**) *ntrBC* and (**B**) *lasI*, but not (**C**) *rhlI* or (**D**) *pqsH*. *P. aeruginosa* LESB58 Δ*ntrBC* strains were seeded with *S. aureus* USA300 at starting OD_600_ = 0.1 in cultures that were sampled in 2-, 6- or 12 h intervals and plated on selective media for bacterial enumeration. Data are presented as mean ± standard error of the mean (SEM) for three independent experiments (*n* = 3).

To validate whether dysregulated expression of QS systems contributed to NtrBC-dependent competitive exclusion of *S. aureus* USA300 LAC by *P. aeruginosa* LESB58, we investigated the competitive phenotype of LESB58 Δ*ntrBC* strains transformed with an overexpression vector containing the coding sequence of genes involved in the synthesis of QS molecules including *lasI*, *rhlI* or *pqsH* (Fig. 6).

As was observed for batch cultures seeded with LESB58 WT and USA300 LAC (Fig. 3A), the density of the complemented LESB58 Δ*ntrBC/ntrBC*^+^ and USA300 increased from the time of inoculation to six h post-inoculation, when USA300 LAC numbers sharply declined from 8.2 × 10^6^ CFU/ml to 5.3 × 10^5^ CFU/ml (Fig. 6A), much as had been observed for WT LESB58 (Fig. 3A). LESB58 Δ*ntrBC/lasI*^+^, when co-cultured with USA300 LAC, showed partial phenotypic complementation, in that the *lasI* overexpressing strain was able to outcompete USA300 LAC at least partially by 12 h post-inoculation (Fig. 6B), with the number of USA300 declining beyond six h post-inoculation, but not to the same extent as observed for LESB58 Δ*ntrBC/ntrBC*^+^*.* In contrast, neither LESB58 Δ*ntrBC/rhlI*^+^ (Fig. 6C) nor LESB58 Δ*ntrBC/pqsH*^+^ (Fig. 6D) were able to outcompete USA300 at any time point. The growth rate of LESB58 Δ*ntrBC/pqsH*^+^ was the lowest of all the strains examined in this mixed species growth experiment, reaching a maximum bacterial density of only 7.9 × 10^6^ CFU/ml around six h post-inoculation, then remaining at this density until the experimental endpoint. Regardless of the LESB58 growth rate, no staphylolytic activity by either of the latter two complemented strains was apparent, since the density of USA300 LAC did not decline at any point (Fig. 6C,D).

**Figure 7:**
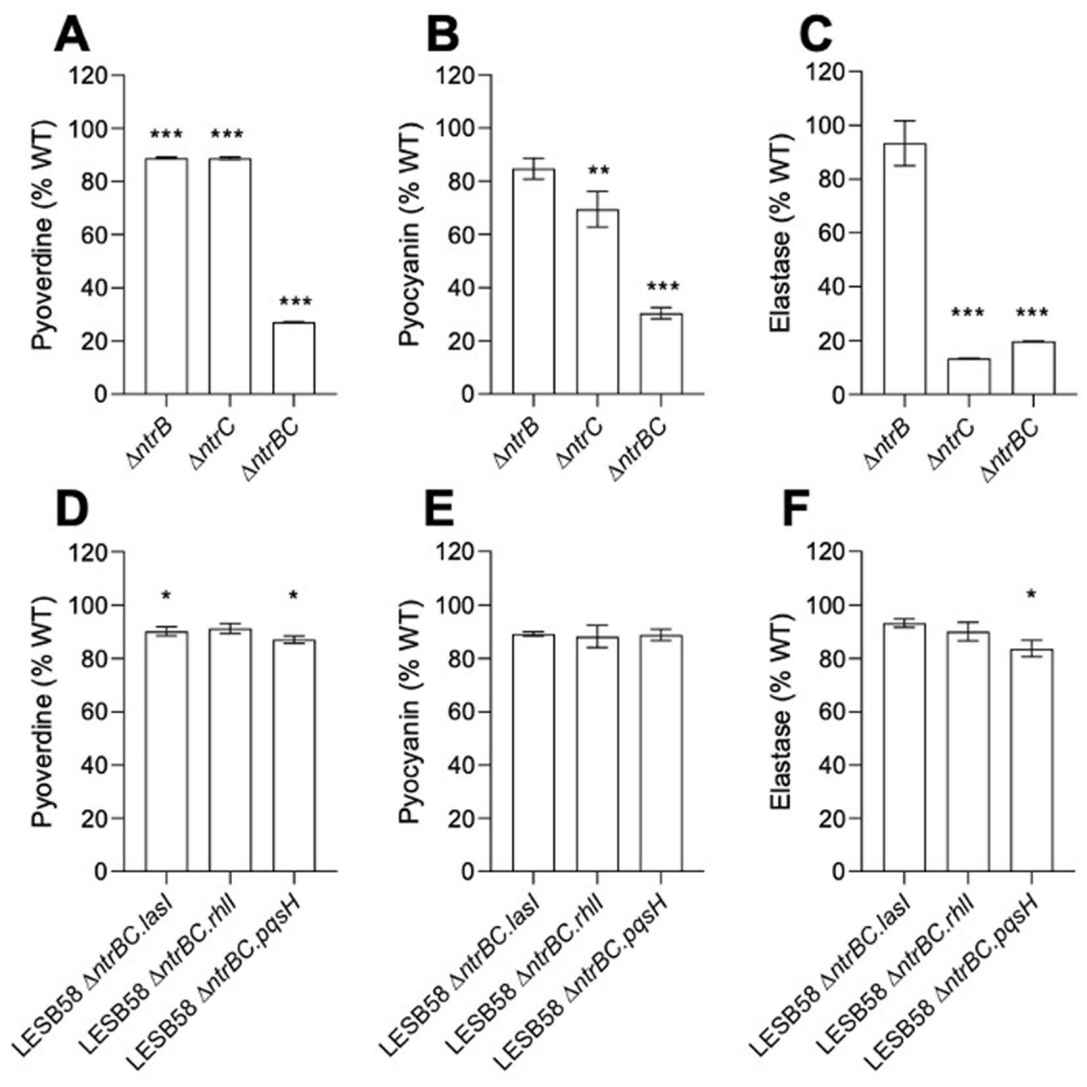
Virulence factor production was restored in *P. aeruginosa* LESB58 Δ*ntrBC* by overexpression of *ntrBC*, *lasI*, *rhlI* and *pqsH*. Levels of (**A,D**) pyoverdine, (**B,E**) pyocyanin and (**C,F**) elastase produced by mutants (**A–C**) or complements (**D–F**) were quantified using well established methods. Data are presented as mean ± standard error of the mean (SEM) for three independent experiments containing three biological replicates in each (*n* = 9). * *P* < 0.05, ** *P* < 0.01, *** *P* < 0.001 different from control according to one-way ANOVA followed by Dunnett’s post-hoc analysis.

To confirm that the overexpression of specific QS determinants phenotypically complemented the decrease in *P. aeruginosa* virulence factors with known anti-Staphylococcal activity^20^, virulence factor secretion was examined in LESB58 strains (Fig. 7). We confirmed previous observations for PA14 mutant strains^16^, in showing that virulence factor production was significantly downregulated in strain LESB58 Δ*ntrBC* and/or Δ*ntrC* strains (Fig. 7A–C), depending on the virulence factor. While statistically significant, reduced production of pyoverdine by LESB58 Δ*ntrB* and Δ*ntrC* was not strong (~ 87% of WT). However, LESB58 Δ*ntrBC* produced only 22% of the level of pyoverdine cf. WT. Similarly, substantial reduction for pyocyanin (to 34% of WT levels) and elastase (19% of WT) was also observed for LESB58 Δ*ntrBC*. LESB58 Δ*ntrB* showed no significant changes in either of these virulence factors, whereas LESB58 Δ*ntrC* produced only 71% (*P* < 0.01), and 17% (*P* < 0.001) as much pyocyanin and elastase as WT. The production of virulence factors by LESB58 Δ*ntrBC* could be restored by overexpression of QS determinants, including *lasI*, *rhlI* and *pqsH* (Fig. 7D–F).

## Discussion

We examined here the importance of NtrBC in interspecies competition between clinical isolates of *S. aureus* and *P. aeruginosa*, pathogens that can be comorbid in upper respiratory tract infections of CF patients, as well as in skin wound infections^2,4^. Induction of the *P. aeruginosa* Liverpool epidemic strain LESB58 *ntrBC* promoter activity was greater in the presence of *S. aureus* USA300 LAC than in monoculture, and this was independent of the ammonium concentration of the supernatant (Fig. 1). Extracellular ammonium is usually correlated with intracellular nitrogen availability of laboratory reference strains of *S. aureus* and *P. aeruginosa*^27,28^ since ammonium is their preferred source of nitrogen. However, clinical isolates may exhibit auxotrophy for essential amino acids, which limits protein synthesis, adaptation and growth, unless that amino acid is abundant in the environment^29,30^. Thus, the extracellular concentration of ammonium may not be the best indicator of intracellular nitrogen status for the strains of *S. aureus* and *P. aeruginosa* used in this study (Fig. 1). However, *ntrBC* can be regulated by other means than through sensing of intracellular nitrogen. Indeed, induction of *ntrBC* promoter activity could be recapitulated by addition of specific *S. aureus* extracellular signaling molecules (Fig. 2), including N-acetylglucosamine, a component of peptidoglycan that can be liberated following bacterial lysis, and D-ribose, an analogue of the autoinducer-2 QS molecule^20^. Future studies could focus on comprehensively examining the secretome of *S. aureus* USA300 LAC during competition and defining the molecular mechanism(s) by which secreted molecules might induce *ntrBC* promoter activity. Interestingly, D-ribose only slightly stimulated *ntrBC* promoter activity in LESB58 WT, but not any mutant strains (LESB58 Δ*ntrB*, Δ*ntrC*, or Δ*ntrBC*) (Fig. 2). This indicated that NtrBC is required for *ntrBC* promoter activity following stimulation by D-ribose during co-culture. While N-acetylglucosamine did not apparently induce *ntrBC* promoter activity in mutant strains (since the amount of luminescence detected at *t* = 0 h was not significantly different from the luminescence detected at later time points in mutant strains), *ntrBC* promoter activity was detected (Fig. 2). This provided evidence that NtrBC was not essential for *ntrBC* promoter activity following stimulation by N-acetylglucosamine. Still, induction of *ntrBC* promoter activity of LESB58 WT was (at its peak) ~ sixfold greater than at the point of inoculation. Taken together, these data indicate that self amplification of NtrBC and/or amplification by exogenous molecules released in co-culture may play a role in NtrBC signaling downstream of interspecies competition with *S. aureus* USA300 LAC. Overall, the data presented (Figs. 1,2) supported our hypothesis that NtrBC was important for conferring a competitive advantage on LESB58 over USA300 LAC, and that NtrBC self amplification of promoter activity, dependent in part on molecules released into the environment by *S. aureus*, may be needed for full responsiveness to interspecies signaling molecules.

*P. aeruginosa* LESB58 WT and Δ*ntrB* strains outcompeted *S. aureus* USA300 LAC in a planktonic competition assay, whereas Δ*ntrC* and Δ*ntrBC* strains did not (Fig. 3). This might be due to crosstalk between NtrC and another sensor kinase, which might activate the regulatory activity of NtrC independently of NtrB, compensating for its deletion. Crosstalk between NtrC and other sensor kinases has been suggested in other bacterial species, including *Escherichia coli*^31^ and *Rhodobacter capsulatus*^32^. Further, NtrC can autophosphorylate in the presence of selected metabolites^33^, bypassing NtrB-mediated activation. Following its activation, NtrC could then regulate the production of anti-Staphylococcal molecules by enhancing RpoN-mediated transcription^12^ or through a mechanism independent of RpoN^13^. The anti-Staphylococcal activity of *P. aeruginosa* in cell-culture systems has been described previously^20^ and attributed to the production of various molecules including 4-hydroxy-2-heptylquinolone N-oxide (HQNO), which is regulated by *Pseudomonas* quinolone signal (PQS), as well as other virulence factors with anti-Staphylococcal activity^20^. Other molecules produced by *P. aeruginosa* that are not typically considered to be virulence factors, such as the acyl homoserine lactone (AHL) molecules involved in the LasRI and RhlRI QS signaling systems, can also interfere with the fitness of *S. aureus* by inhibiting respiratory (electron transport chain) activity and preventing planktonic growth^20^. The anti-Staphylococcal activity of *P. aeruginosa* is known to be influenced by environmental factors, including the presence of host factors such as serum or mediators of immune signaling^20^. Accordingly, it has been observed that competitive inhibition by *P. aeruginosa* of *S. aureus* can be muted in host-like conditions characteristic of, for example, animal models of disease and biofilm formation in host-mimicking media^3,34^. This could partially explain why different patterns of competitive exclusion were exhibited by strains of *P. aeruginosa* LESB58 (WT, Δ*ntrB*, Δ*ntrC*, or Δ*ntrBC*) in biofilm assays in vitro (Fig. 4A–B) and in an air–liquid interface skin organoid model (Fig. 4C–D). Generally, LESB58 and USA300 LAC co-existed better during in vitro biofilm growth, suggesting that either *S. aureus* USA300 LAC was producing fewer molecules that primed *P. aeruginosa* LESB58 strains and/or the latter demonstrated muted production of anti-Staphylococcal molecules.

To further explore this issue, the mechanism(s) possibly underlying inhibition of *S. aureus* USA300 LAC by different strains of *P. aeruginosa* LESB58 were interrogated in the context of planktonic or biofilm competition assays. Upstream regions of coding sequences of *P. aeruginosa* LESB58 were searched for NtrC binding motifs (Fig. S2), by inputting a prior defined position weight matrix^22^ to FIMO software^23^, identifying the potential binding locations (Table S4). The number of hits identified was likely an underestimate, since the binding motif of NtrC is not well conserved, and since NtrC is known to bind to RpoN directly from solution^35^, making it more challenging to identify members of the NtrBC regulon by this approach. Leads identified by FIMO included the alternative sigma factor PvdS^36^, implicated in iron scavenging and pyoverdine synthesis for iron acquisition as well as in exotoxin A production, and expression of the transcriptional regulator LasR^37^, the master regulator of the hierarchical QS regulatory network of *P. aeruginosa*. Differential expression of strong leads was confirmed by RT-qPCR (Table 1). Although *P. aeruginosa* LESB58 mutants exhibited different competitive phenotypes when co-cultured with *S. aureus* USA300 LAC (Fig. 3), differential expression of selected genes encoding anti-Staphylococcal molecules was similar across strains (Table 1). This could be due to the different experimental conditions used to examine competition and genetic regulation, or could indicate that expression of other anti-Staphylococcal molecules that might be impacted during co-culture and may have contributed to the observed phenotypes. Additionally, downregulated expression of *phzA1* (Table 1), which encodes a phenazine biosynthetic protein, did not always correlate with lesser production of the phenazine pyocyanin (Fig. 7). Since PhzA1 is involved in the synthesis of phenazine-1-carboxylic acid, which is further oxidized to pyocyanin or one of three other phenazines, and there are two functionally redundant operons for this process encoded in *P. aeruginosa*, it is difficult to determine exactly why this was. Nonetheless, LasR was considered a strong lead due to its expression from an RpoN-dependent promoter^34^, global regulation of processes including synthesis of virulence factors with anti-Staphylococcal activity^20^, and differential expression in PA14 Δ*ntrB* and Δ*ntrC* mutants^15^. Thus, the impact of overexpression of QS molecules on competitive and virulence phenotypes of LESB58 Δ*ntrB,* Δ*ntrC* and Δ*ntrBC* mutants, was examined (Figs. 6 and 7). Overexpression of *lasI* in the LESB58 Δ*ntrBC* genetic background restored competition with USA300 (Fig. 6), as also reflected by the restoration of pyoverdine, pyocyanin and elastase production (Fig. 7). However, *rhlI* and *pqsH* did not restore the competitive advantage of LESB58 Δ*ntrBC* (Fig. 6)*,* despite improving pyoverdine, pyocyanin and elastase production (Fig. 7). This indicated that other anti-Staphylococcal molecules, such as N-dodecanoyl-L-homoserine lactone, might be regulated by something downstream of the LasRI QS system, but not other QS systems lower in the hierarchical QS regulatory network.

## Materials and methods

### Bacterial strains and growth conditions

Bacterial strains and plasmids used in this study are described in Table S1. Overnight cultures were routinely maintained in Luria–Bertani (LB) broth or 2 × yeast extract tryptone (2xYT) prepared according to the manufacturer’s specifications (Thermo Scientific). Overnight and sub-cultures were incubated for no longer than 18 h at 37 °C with shaking (250 rpm). Modified forms of basal medium (BM2; containing 62 mM potassium phosphate buffer (pH = 7.0), 0.1% casamino acids (CAA) and/or 7 mM (NH_4_)_2_SO_4_, 2 mM MgSO_4_, 10 μM FeSO_4_, 20 mM glucose) were used for promoter induction assays, competition assays and biofilm induction assays in vitro. Other media used in specific assays are described elsewhere. Gentamicin (500 μg/ml) was added to growth media for plasmid selection in *P. aeruginosa* LESB58 strains. Kanamycin (30 μg/ml) or gentamicin (15 μg/ml) was added to growth media for plasmid selection in *Escherichia coli* DH5α. Bacterial growth was monitored by measuring optical density (OD_600_) with a spectrophotometer (Eppendorf, Missisauga, Canada).

### Generation of bioluminescence reporter strains

High-fidelity polymerase chain reaction (PCR) was carried out using the Phusion DNA Polymerase (Thermo Scientific) in accordance with the manufacturer’s specifications and optimized annealing temperatures. Oligomer sequences were based on the genome of *P. aeruginosa* LESB58 (GenBank: NC_002516.2) available from NCBI. For colony PCR reactions performed on LESB58, cells were boiled at 98 °C with shaking (1,000 rpm) for 10 min and pelleted by centrifugation at 14,500 rpm for 3 min. Restriction digests were performed using FastDigest restriction enzymes according to the manufacturer’s specifications (Thermo Scientific). All ligation reactions were carried out at room temperature using T4 DNA ligase (Invitrogen). DNA purifications were performed using the GeneJET PCR purification kit or the GeneJET Gel extraction kit following the manufacturer’s instructions (Thermo Scientific).

To generate recombinant strains, the coding sequences of LESB58 *rhlI*, *lasI* and *pqsH* were PCR amplified, gel purified and digested with restriction enzymes *EcoRI* and *BamHI*. PCR products were subsequently cloned into *EcoRI*/*BamHI* digested pBBR1MCS-5. LESB58 were scraped from an agar plate and resuspended in 300 mM sucrose. After washing twice, pelleted cells were resuspended in 100 µl of 300 mM sucrose and mixed with 500 ng of plasmid. Cells were transformed via electroporation (2.5 kV, 25 μF, 200 Ω). All steps were carried out at room temperature. Cells were recovered for 3 h at 37 °C in 2xYT broth with shaking at 220 rpm after electroporation.

Plasmid pUC18T-min-Tn7T-lux^38^ was modified by cloning the *EcoRI*/*BamHI* digested *ntrBC* promoter into the multiple cloning site. The derivative pUC-Tn7T-lux-*ntrBC* was co-electroporated with helper plasmid pTNS2^39^ into electrocompetent *P. aeruginosa* LESB58 strains, as described above. Positive clones, showing strong bioluminescence, were selected on LB agar plates containing gentamicin and further verified for correct chromosomal insertion via PCR of the flanking regions with transposon- and chromosome-specific primers as described previously^39,40^.

### Promoter induction assays in vitro

Luminescently tagged bacteria were seeded at a density of ~ 1.5 × 10^7^ CFU/ml in flat-bottomed 96-well white plates (Corning) containing BM2 with or without signaling molecules. Plates were incubated at 37 °C with continuous shaking (250 rpm). OD_600_ and luminescence measurements were taken in one h increments for 20 h (Synergy H1, BioTek). Experiments were performed three times with at least three technical replicates. The ammonium concentration in the medium was measured, in parallel, using an ammonia assay kit (Sigma) on centrifuged (8000 rpm for 5 min) and filtered (0.2 μm pore size) cell supernatants, according to the manufacturer’s specifications.

### Competition assays

Each species of bacteria was seeded at an adjusted OD_600_ = 0.1 in batch cultures. Competition assays with LESB58 and USA300 LAC were grown for 24 h, with shaking (250 rpm) at 37 °C. Samples were taken for serial dilution and bacterial enumeration on selective media (mannitol salt agar (MSA) and *Pseudomonas* isolation agar (PIA) prepared according to the manufacturer’s specifications) after 18–34 h incubation. Experiments were performed three times.

### Biofilm formation in vitro

Biofilm assays were performed as previously described^41^, with minor modifications. Briefly, bacteria were scraped from a plate, resuspended in phosphate buffered saline (PBS) (pH = 7.4, Gibco) and mixed at OD_600_ = 0.1. Polymicrobial cultures were seeded into round-bottomed 96-well polypropylene plates (Corning) and incubated at 37 °C for 24–28 h. Planktonic cells were removed and biofilms were washed prior to staining with crystal violet (0.1%) or resuspension and serial dilution for bacterial enumeration on selective media (MSA and PIA) after overnight incubation at 37 °C. Experiments were performed three times with three technical replicates in each.

### Biofilm formation on a human skin organoid model

A human air liquid interface organoid model^42^ was modified by using Ker-CT human keratinocytes (ATCC CRL_4048) that were routinely cultured in Keratinocyte-SFM medium (Gibco) at 37 °C, 5% CO_2_. Human skin-equivalent organoids were formed by seeding cells on Transwell filter inserts (0.4 μm pore size) in deep 12-well ThinCert™ plates containing DermaLife K Keratinocyte Complete Medium (Lifeline Cell Technology) prepared according to the manufacturer’s specifications. After growth to confluency the medium in the Transwells above the keratinocyte layer was removed for 2–3 days to initiate multi-structured skin formation^42^. Prior to infection, K0 medium (Dulbecco’s Modified Eagle Medium supplemented with Ham’s F-12 (hydrocortisone, isopreterenol, insulin, selenious acid, L-serine and L-carnitine; Gibco) was added to the wells.

Bacteria from overnight cultures were sub-cultured in BM2 with 0.1% CAA to mid-log phase (OD_600_ = 0.4–0.6) prior to infection. Bacteria were then washed twice in PBS and resuspended to an OD_600_ = 0.1 for each species. Polymicrobial cultures were then added to the apical surface of the human skin organoid model for biofilm formation. Infected skin organoids were incubated at 37 °C, 5% CO_2_ for 24 h. Uninfected controls were treated with Triton X-100 (Sigma) and skins were incubated for an additional one h. Transfer inserts were removed from wells and skins were extracted for homogenization followed by serial dilution and enumeration of colony forming units (CFU/ml) on selective media (MSA and PIA) after overnight incubation at 37 °C. Supernatants were tested for lactate dehydrogenase (LDH) release due to cell lysis using an LDH assay kit as previously described^43^. Experiments were performed three times with one or two technical replicates.

### Bacterial colonization in mice

Animal experiments were performed in accordance with the Canadian Council on Animal Care (CCAC) guidelines and were approved by the University of British Columbia Animal Care Committee (protocol A19-0064). The study is reported in accordance with ARRIVE guidelines 2.0^44^. Mice used in this study were inbred CD-1 mice (female, aged 5–7 weeks). No mice were excluded from analysis. All animals were purchased from Charles River Laboratories, Inc. (Wilmington, MA) and underwent a one-week acclimatisation period in the Modified Barrier Facility at UBC. CD-1 mice weighed 25 ± 5 g at the time of experiment and were group housed in cohorts of 4–5 littermates exposed to the same bacterial strains. Otherwise, to minimise potential confounders, order of treatment and examination of mice was done randomly. Blinding was not used at any step of data collection due to isolated working conditions under COVID-19 safety protocols in the animal facility. Standard animal husbandry protocols were employed.

Bacterial colonization in vivo was assessed using a subcutaneous abscess model, as previously described^21^. Briefly, luminescently-tagged LESB58 and non-luminescent USA300 LAC subcultures were grown to stationary phase, then washed twice with sterile PBS and resuspended at an OD_600_ = 1.0. For monoculture assays, only LESB58 (50 μl) was inoculated. For co-culture assays, species were mixed and 50 μl were injected subcutaneously into the right dorsum of mice. Bacterial density of inocula were constant. Abscesses were formed for 72 h prior to measurement of visible dermonecrosis, a primary outcome. Luminescence, another primary outcome, was monitored in 24 h increments using an in vivo imaging system (IVIS; Perkin Elmer, Waltham, MA, USA). Luminescence from mice inoculated with mixed species was compared to luminescence from mice inoculated with LESB58 only. Abscesses were harvested for bacterial enumeration on selective media (MSA and PIA) following homogenization and serial dilution. Number of bacteria recovered and size of abscesses from mixed species abscesses were compared to WT. Experiments were repeated three times with two replicates in each, and a total of 36 mice were used.

### Virulence factor production assays

Pyoverdine was assessed as previously described^45^. Briefly, bacteria were incubated in Casamino acid medium (0.5% CAA, 0.1 mM MgSO_4_, 0.4% glucose, 7 mM potassium phosphate buffer, pH = 7.0) at 37 °C (250 rpm). Turbid cultures were pelleted, and the supernatant was collected in a fresh microfuge tube. Five μl of supernatant was mixed with 995 μl 10 mM Tris–HCl (pH = 6.8). Pyoverdine was quantified based on intrinsic fluorescence at an excitation wavelength of 400 nm and emission 460 nm using a microplate reader (Synergy H1, Biotek). Pyocyanin concentrations were determined spectrophotometrically after extraction with chloroform and 0.2 M HCl as described elsewhere^46^. Absorbance at 520 nm was read (Synergy H1, Biotek). Elastase was determined by proteolysis of Elastin-Congo red complex (Sigma) as described elsewhere^47^. Five hundred μl of supernatant from cultures grown for 16 h was collected, added to 10 mg/ml Elastin-Congo red in PBS (pH = 7.4) and incubated at 37 °C (250 rpm) for eight h. Absorbance of the aqueous fraction was examined at 495 nm (Synergy H1, Biotek). Experiments were performed three times with three biological replicates in each.

### Transcriptomic studies

LESB58 strains were sub-cultured to an OD_600_ = 0.4–0.6 and spot cultured onto BM2 glucose agar plates for 18–24 h at 37 °C. Surface colonized cells were harvested from the plate in PBS containing RNAProtect (at a 1:2 ratio) reagent (Qiagen). RNA extraction from three biological replicates was performed using the RNeasy Mini Kit (Qiagen) according to the manufacturer’s specifications. Deoxyribonucleases were removed using the TURBO DNA-free kit (Thermo Fisher). RT-qPCR was used to validate expression of selected dysregulated genes previously identified in the mutants using RNA-Seq^15^. Reaction samples were prepared using qScript one-step SYBR green RT-qPCRKit (QuantaBio) with 0.2 ng/μl RNA. Amplification was performed using a LightCycler 96 instrument (Roche, Indianapolis, IN). Gene expression was quantified by the ΔΔC_t_ method with normalization to *rpoD* expression^48^. Primers used for qRT-PCR are listed in Table S2.

### Binding site analysis

To predict sites where NtrC or RpoN directly bound to DNA, a position weight matrix (PWM) model was generated from available ChIP-Seq or HT-SELEX data using Autoseed software and manual refinement^49,50^. Sites upstream of coding sequences in the LESB58 genome were scanned for binding sites using the Find Individual Motif Occurrences (FIMO) software^23^ that returned significant hits with *P* < 10^–4^.

### Statistical analysis

Statistics were performed using GraphPad Prism 9.0 (La Jolla, CA, USA). *P* values were calculated using One-Way or Two-Way analysis of variance (ANOVA) with post-hoc analysis as indicated in the Figure captions. Statistical significance was established when *P* < 0.05.

## Supplementary Information

Supplementary Information.

## Data Availability

Datasets discussed in this manuscript are publicly accessible in the NCBI Gene Expression Omnibus (GEO) database under the accession number GSE145591.
