# Supplementary material for: Competition between Pseudomonas aeruginosa and Staphylococcus aureus is dependent on intercellular signaling and regulated by the NtrBC two-component system
Source: Sci Rep. 2022 May 30;12:9027. doi: 10.1038/s41598-022-12650-2 (PMC9150766; doi:10.1038/s41598-022-12650-2)
Supplement: Supplementary file 1 — Supplementary Information. [file 41598_2022_12650_MOESM1_ESM.pdf]

Competition between *Pseudomonas aeruginosa* and *Staphylococcus aureus* is dependent on intercellular signaling and regulated by the NtrBC two-component system

Morgan A. Alford, Simranpreet Mann, Noushin Akhoundsadegh, Robert E.W. Hancock

Centre for Microbial Diseases and Immunity Research and Department of Microbiology, University of British Columbia, Vancouver, BC, Canada

*** Correspondence:**
Robert E.W. Hancock, e-mail: bob@hancocklab.com

Supplemental information

Table S1. Bacterial strains and plasmids used in this study.

Strain	Characteristics	Reference
Pseudomonas aeruginosa		
LESB58	WT *P. aeruginosa* Liverpool Epidemic Strain B58	[51]
LESB58 Δ*ntrB*	LESB58 *ntrB* chromosomal deletion	[15]
LESB58 Δ*ntrC*	LESB58 *ntrC* chromosomal deletion	[15]
LESB58 Δ*ntrBC*	LESB58 *ntrBC* chromosomal deletion	[15]
LESB58 *ntrBC*-pro	LESB58 *ntrBC*-pro-*luxCDABE*	This study
LESB58 Δ*ntrB ntrBC*-pro	LESB58 Δ*ntrB*, *ntrBC*-pro-*luxCDABE*	This study
LESB58 Δ*ntrC ntrBC*-pro	LESB58 Δ*ntrC*, *ntrBC*-pro-*luxCDABE*	This study
LESB58 Δ*ntrBC ntrBC*-pro	LESB58 Δ*ntrBC*, *ntrBC*-pro-*luxCDABE*	This study
Staphylococcus aureus		
USA300 LAC	Community-acquired MRSA, parental strain	[52]
Escherichia coli		
DH5α	F^- Φ80*lac*ZΔM15 U169 *rec*A1 *end*A1 *hsd*R17(r_k^-, m_k^+) *pho*A *sup*E44 *thi*-1 *gyr*A96 *rel*A1 λ^-	Thermo Fisher
**Plasmids**		
pTNS2	pTNS::*lacI*-Pro::RSF, Kan^r	[36]
pUC18T-min-Tn7T-lux	pUC18T::mini-Tn7T::*luxCDABE*::*P1*-Pro, Gm^r	[35]
pUC-Tn7T-lux-*ntrBC*	Derivative of pUC18-mini-Tn7T-lux, cloned 0.5 kbp *ntrBC*-pro, Gm^r	This study
pBBR1MCS-5	Broad host-range cloning vector, Gm^r	[53]
pBBR.*lasI*	Cloned 0.606 kbp *lasI* gene, Gm^r	This study
pBBR.*rhlI*	Cloned 0.606 kbp *rhlI* gene, Gm^r	This study
pBBR.*pqsH*	Cloned 1.15 kbp *pqsH* gene, Gm^r	This study

Table S2. Primers used in this study.

Primer name	Sequence (5’ -> 3’)
**Complementation primers**	
lasI-fwd	TTTCGCCATCAACTCTGGACA
lasI-rev	CGTACAGTCGGAAAAGCCCA
rhlI-fwd	GTCCATGGCACCTATCCCAA
rhlI-rev	AGACCACCATTTCCGAGGAG
pqsH-fwd	GGGGGGCCCGGTTACCTCTTGCACGCGA
pqsH-rev	GATAAGCTTAGCGACCATCGCCGAAGTC
**RT-qPCR primers**	
phzA1-fwd	TAAAACGTAATCGCGAGTTCATG
phzA1-rev	TTTTATTTGCGGAACGGCTATT
pyS2-fwd	GCAGCACAAAGTCACCGAAGG
pyS2-rev	CCGTGGGAAACCACTTCAGC
algU-fwd	GATATCGACAATGCCGTTCC
algU-rev	CAGGACTTGCGCTTCTTCTT
pvdS-fwd	GGCAAGTGGGAGGTGAACTA
*pvdS*rev	AAGTACTTGCGCACCGTCTC
lasR-fwd	TCACATTGGCTTCCGAGCAG
lasR-rev	AAACCGGTGGTTCTGACCAG
plcH-fwd	CATGGAATGGGTCAACCTGT
plcH-rev	AGATCGAGGCGTTCTTCTTG

Table S3. Growth constants for LESB58 wild-type (WT) and mutant (Δ*ntrB*, Δ*ntrC*, Δ*ntrBC)* strains co-cultured with USA300 LAC during exponential growth phases. The natural log of the number of cells at the end of exponential growth minus the natural log of the number of cells at the beginning of exponential growth equals the growth rate constant multiplied by the time interval.

Strain	t_n	t_0	Bacterial growth rate constant (*μ*)
LESB58 WT	12 h	2 h	0.43/h
LESB58 Δ*ntrB*	12 h	2 h	0.40/h
LESB58 Δ*ntrC*	12 h	2 h	0.21/h
LESB58 Δ*ntrBC*	12 h	4 h	0.12/h

Table S4. FIMO software detected 259 NtrC binding targets in the promoter regions of *P. aeruginosa* coding sequences. Only nonredundant binding sites are shown, with Start and Stop delineating how many bp upstream of the start codon NtrC bound. FIMO calculated the *P* and *Q* values for each binding event. The matched sequence corresponding to the NtrC binding motif is shown. Additionally, the FC expression of genes downstream of NtrC binding sites that were DE in *P. aeruginosa* PA14 Δ*ntrB* or Δ*ntrC* [15] according to RNA-Seq

Name	Start	Stop	Score	*P*	*Q*	Matched Sequence	FC Δ*ntrB*	*FC ΔntrC*
cheA	186	200	10.3	9.8E-05	0.66	CCGAACATTCCGCTG		
moaE	153	167	10.3	9.8E-05	0.66	TCGAACATTATCCGG		
PA14_14430	277	291	10.3	9.6E-05	0.65	TAGAATAATTTTCCT		
PA14_22075	23	37	10.3	1.0E-04	0.66	TGAAACATCCTGCTG		
PA14_26150	28	42	10.3	1.0E-04	0.66	CGGTAGATTGTGCCA		
PA14_26160	24	38	10.3	1.0E-04	0.66	CGGTAGATTGTGCCA		
PA14_29590	188	202	10.3	9.8E-05	0.66	AACTATATTGTTCTT		
PA14_52890	150	164	10.3	1.0E-04	0.66	CAGAAGATCAACCTG		
PA14_54690	35	49	10.3	1.0E-04	0.66	CGGGATAAACCGCTG		
PA14_54830	383	397	10.3	9.8E-05	0.66	GAGCATTTTGTATTC	-6.4	-7.7
alkA	221	235	10.4	9.0E-05	0.64	CAGGGCGAAATGCT		
aprA	148	162	10.4	9.2E-05	0.64	AAAGATAAACTACCG		
ccmE	94	108	10.4	8.9E-05	0.64	CAGAACATCAACCTG		
flgF	119	133	10.4	9.1E-05	0.64	TAAATCAATGTATTA	-2.7	-2.1
hprA	190	204	10.4	9.3E-05	0.64	AAATATGTAATACCG		
ilvI	248	262	10.4	9.0E-05	0.64	TCAAGCTTTATTCTG		
PA14_14430	150	164	10.4	8.9E-05	0.64	TAAAGAAATATTTTG		
PA14_16960	73	87	10.4	8.9E-05	0.64	CCGAACAACCTGCTG		
PA14_17000	307	321	10.4	9.4E-05	0.64	CAGTGCGTTCTGCTG		
PA14_28280	222	236	10.4	9.2E-05	0.64	GAGAACAATCTTTCG	-1.6	-1.6
PA14_48140	694	708	10.4	9.3E-05	0.64	CAGTTCAATACTTTG		
PA14_48150	278	292	10.4	9.3E-05	0.64	CAAAGTATTGAACTG		
PA14_54860	12	26	10.4	9.0E-05	0.64	TCGAACGTTGTTCTA		
PA14_61200	117	131	10.4	9.3E-05	0.64	AAATATGTAATACCG		
PA14_62170	289	303	10.4	9.0E-05	0.64	TCAAGCTTTATTCTG		
PA14_63230	112	126	10.4	9.3E-05	0.64	AAAGACTTCATTTTA		
PA14_63240	29	43	10.4	9.3E-05	0.64	AAAGACTTCATTTTA		
PA14_65840	331	345	10.4	9.3E-05	0.64	CAGTGCATTGCGCTG		
PA14_66410	33	47	10.4	8.9E-05	0.64	CCGAGCATTGTGCTG		
ptsP	164	178	10.4	9.0E-05	0.64	CAGGACGAAACGCT		
aceE	97	111	10.5	8.1E-05	0.63	TAAAACTACATTTCG		
argC	1	15	10.5	8.1E-05	0.63	CAAGAGGATGTCCTG		
glnE	171	185	10.5	8.1E-05	0.63	TAAAACTACATTTCG		
gntR	167	181	10.5	8.2E-05	0.63	CCAAACAAAATTCCA		
oprP	166	180	10.5	8.8E-05	0.64	CAAGAGTTTCTGCCG		
PA14_02450	226	240	10.5	8.6E-05	0.64	CAAAGCAAAACTTTG	-1.9	-2.9
PA14_08470	276	290	10.5	8.1E-05	0.63	CAAGAGGATGTCCTG		
PA14_12890	106	120	10.5	8.7E-05	0.64	CAGGACGACATCCTG		
PA14_30460	176	190	10.5	8.2E-05	0.63	TATCTTAATATGCTA		
PA14_34640	14	28	10.5	8.2E-05	0.63	CCAAACAAAATTCCA		
PA14_43430	32	46	10.5	8.2E-05	0.63	TGAAACTATATTTCG		

PA14_43440	196	210	10.5	8.2E-05	0.63	TGAAACTATATTTCG		
PA14_48140	539	553	10.5	8.3E-05	0.63	AAGGGTTATATCCTT		
PA14_48150	433	447	10.5	8.3E-05	0.63	AAGGGTTATATCCTT		
PA14_48460	64	78	10.5	8.8E-05	0.64	GAAGAGAAAGTGCTG		
PA14_51550	204	218	10.5	8.8E-05	0.64	AAACGGATTCTACTA		
PA14_53700	29	43	10.5	8.2E-05	0.63	GCGCATAATATTTCG		
PA14_53720	58	72	10.5	8.2E-05	0.63	GCGCATAATATTTCG		
PA14_59630	53	67	10.5	8.5E-05	0.63	CAGTAGGAAATACTC		
PA14_59640	544	558	10.5	8.5E-05	0.63	CAGTAGGAAATACTC		
atpB	21	35	10.6	7.6E-05	0.63	AAGCGGTTTCTGCTG		
PA14_07430	528	542	10.6	7.6E-05	0.63	CATAACAATATGCCG		
PA14_18960	230	244	10.6	7.9E-05	0.63	TAAAAAATCATACTG		
PA14_19480	55	69	10.6	7.9E-05	0.63	AAAAAGAATAAATTA		
PA14_19490	137	151	10.6	7.9E-05	0.63	AAAAAGAATAAATTA	2.1	2.3
PA14_27980	97	111	10.6	7.8E-05	0.63	CAAAAATAAATATTG		
PA14_27990	192	206	10.6	7.8E-05	0.63	CAAAAATAAATATTG		
PA14_44950	502	516	10.6	7.8E-05	0.63	CAAAGTAATTTTTTG		2.0
PA14_64530	124	138	10.6	7.9E-05	0.63	TAATTGATTATTTTT		
PA14_72370	459	473	10.6	8.0E-05	0.63	GAAAACATCATGCTT		
pcoA	257	271	10.6	8.0E-05	0.63	GAGCAGGTTCTGCTG	-2.1	-4.0
plcH	58	72	10.6	7.6E-05	0.63	TCAAATAAAATGTGA		
pscR	139	153	10.6	7.9E-05	0.63	CAGCAGATTCCGCCG	12.6	20.7
pyrG	66	80	10.6	7.5E-05	0.63	CAAAGCAAACTCCTG	1.8	
rpoD	24	38	10.6	7.8E-05	0.63	CAGAGGATTATACCT		
rpsI	6	20	10.6	8.1E-05	0.63	TAGCTCATTATGTCG		
murG	4	18	10.7	7.1E-05	0.62	CAGGACATTACCTTT		
PA14_01840	76	90	10.7	6.9E-05	0.62	CAAATGATAATGCTT		-3.3
PA14_08650	91	105	10.7	7.4E-05	0.63	AAGAGTTTTCTCTTA		
PA14_15830	68	82	10.7	7.3E-05	0.63	TAAAAGAAAACGCCG		
PA14_34320	161	175	10.7	7.1E-05	0.62	CAACAGTTTTTGCTG		
PA14_34330	244	258	10.7	7.1E-05	0.62	CAACAGTTTTTGCTG		
PA14_53570	210	224	10.7	7.1E-05	0.62	GAGCAGATCGTGCTG		
PA14_53580	135	149	10.7	7.1E-05	0.62	GAGCAGATCGTGCTG		
PA14_61320	200	214	10.7	7.0E-05	0.62	CAGAGGGTTCTGCTG		
PA14_61330	212	226	10.7	7.0E-05	0.62	CAGAGGGTTCTGCTG		
PA14_63120	252	266	10.7	7.2E-05	0.62	CAGCACGTTCTGCCA		
PA14_68530	21	35	10.7	7.0E-05	0.62	CAAGACTAAATTTCA	-2.6	
PA14_68550	108	122	10.7	7.0E-05	0.62	CAAGACTAAATTTCA		
pcnB	252	266	10.7	7.3E-05	0.63	CAAAAGAATCAGTTA		
glnA	41	55	10.8	6.3E-05	0.61	TCAGATATAATTCCG		-2.0
metN	21	35	10.8	6.8E-05	0.61	AAGCGCTTTCTGCTG		
mexC	77	91	10.8	6.6E-05	0.61	CAAAAGATCATTTGA		
nfxB	70	84	10.8	6.6E-05	0.61	CAAAAGATCATTTGA		
PA14_02390	237	251	10.8	6.5E-05	0.61	TAGAAAAATATGCGA		
PA14_02410	100	114	10.8	6.5E-05	0.61	TAGAAAAATATGCGA		
PA14_13670	7	21	10.8	6.8E-05	0.61	CAGGAGATACCGCTA		
PA14_24770	326	340	10.8	6.5E-05	0.61	CAACAGTTCATCCTT		2.1
PA14_27980	82	96	10.8	6.3E-05	0.61	AAGAATTTTATAGTT		
PA14_27990	207	221	10.8	6.3E-05	0.61	AAGAATTTTATAGTT		
PA14_28990	83	97	10.8	6.8E-05	0.61	CAGATTTTTCTGTTT		
PA14_34880	123	137	10.8	6.3E-05	0.61	TAACAAGTTATGCTG		

PA14_45250	284	298	10.8	6.3E-05	0.61	CAATTGATTCTGCTG		
PA14_56910	184	198	10.8	6.6E-05	0.61	AAGGACATCCTGCTG		
pcrD	43	57	10.8	6.6E-05	0.61	AAGGACATCCTGCTG	2.7	3.8
thiI	282	296	10.8	6.3E-05	0.61	TCAGATATAATTCCG		
aph	199	213	10.9	6.0E-05	0.61	CAGCACTTCCTGCTT		
argF	20	34	10.9	6.2E-05	0.61	CGACGCTTTATGCTG		
dtd	57	71	10.9	5.8E-05	0.61	AAAAACTTTATTTGA		
oprE	79	93	10.9	6.1E-05	0.61	AGGAATATTGTACCG	1.8	1.8
PA14_00970	151	165	10.9	6.1E-05	0.61	CAGAACAACCCGCTG		
PA14_17250	64	78	10.9	6.0E-05	0.61	CAGGACTTTCCGCTA		
PA14_18620	245	259	10.9	6.2E-05	0.61	CAGCATAAAGCGTCG		
PA14_19170	219	233	10.9	6.0E-05	0.61	TAGGGGAATACGCTG	1.8	1.8
PA14_19190	225	239	10.9	6.0E-05	0.61	TAGGGGAATACGCTG		
PA14_22210	19	33	10.9	6.0E-05	0.61	AAACACAAAATCCCG		
PA14_23420	10	24	10.9	6.2E-05	0.61	CAAAATATAAAACCA		
PA14_26450	753	767	10.9	5.9E-05	0.61	CGGGATAATCTTCGG		
PA14_48140	397	411	10.9	5.8E-05	0.61	CATCACAAAATATTG		
PA14_48150	575	589	10.9	5.8E-05	0.61	CATCACAAAATATTG		
PA14_49030	344	358	10.9	6.0E-05	0.61	CAACATTATAAACCG		
PA14_52000	11	25	10.9	6.0E-05	0.61	AAGAACAAACCGCTG		
PA14_59220	21	35	10.9	6.1E-05	0.61	CAGCAGGAAATACTC		
PA14_59230	53	67	10.9	6.1E-05	0.61	GAGTATTTCCTGCTG		
PA14_71430	51	65	10.9	6.0E-05	0.61	AAAGACAAAATTCTC		
pilL2	205	219	10.9	6.1E-05	0.61	CAGCAGGAAATACTC		
betT1	450	464	11	5.5E-05	0.61	CAGCATGTAGTACCA		
lasR	155	169	11	5.3E-05	0.61	TCACATTTTATGCGA		
nrdA	108	122	11	5.4E-05	0.61	AAACACTACATATTG		
PA14_05500	108	122	11	5.5E-05	0.61	CAGAAAATTCTGCTT		
PA14_10940	363	377	11	5.7E-05	0.61	CAGGGGAATGTCCTG		
PA14_12570	289	303	11	5.3E-05	0.61	TAAAAGAATACGTCG		
PA14_13940	246	260	11	5.4E-05	0.61	CAAGAGTATCTCTTA		
PA14_19530	117	131	11	5.6E-05	0.61	ACGAATATTCTGTTT		
PA14_20510	688	702	11	5.3E-05	0.61	CAATAGAAAACCTTG		
PA14_21020	259	273	11	5.5E-05	0.61	TAACTGTTAATTCTG		
PA14_21030	209	223	11	5.5E-05	0.61	TAACTGTTAATTCTG		
PA14_31100	341	355	11	5.4E-05	0.61	CAGCACGACGTGCTG		
PA14_49440	611	625	11	5.4E-05	0.61	AAACACTACATATTG		
PA14_53620	1052	1066	11	5.7E-05	0.61	GAGAAGAACGTTCTG		
PA14_54610	25	39	11	5.5E-05	0.61	CAAAACAACATCCCG		
PA14_54620	64	78	11	5.5E-05	0.61	CAAAACAACATCCCG		
PA14_68470	134	148	11	5.7E-05	0.61	CAGAACATTGCTCCA		
PA14_68480	76	90	11	5.7E-05	0.61	CAGAACATTGCTCCA		
pcrV	94	108	11	5.3E-05	0.61	CAGGAGGAACTGCT	4.5	7.7
sndH	127	141	11	5.4E-05	0.61	CAGAAGGAAGTGCTT		4.6
speD	114	128	11	5.7E-05	0.61	CGGAAGAATCTTCCG		
PA14_02930	164	178	11.1	5.0E-05	0.61	TCAAATAATCTCCTT		
PA14_28370	359	373	11.1	5.1E-05	0.61	CAAAACTTCATTCCA		-1.9
PA14_29750	31	45	11.1	5.1E-05	0.61	CAGGAGGAAATCTTG		
PA14_48760	409	423	11.1	5.2E-05	0.61	AAGAACAACGTCCTG		
PA14_49040	314	328	11.1	4.8E-05	0.61	TAGAAGTTTAAGCTT		
PA14_62690	152	166	11.1	5.1E-05	0.61	AAAAACAATAAGTTA		3.0

pyrH	235	249	11.1	5.1E-05	0.61	TAGAAGAATGCGTTT	1.6	
algU	496	510	11.2	4.5E-05	0.61	CAGTATCTTGTGCTG		2.8
metN	112	126	11.2	4.8E-05	0.61	CAAGGTAAAATGCTC		
modA	28	42	11.2	4.5E-05	0.61	TAGCGCTATATTCCA		
osmE	227	241	11.2	4.7E-05	0.61	CAGGACTTCCTGCTG	1.9	3.7
PA14_14280	235	249	11.2	4.8E-05	0.61	CAGCTTGATCTGCTG		
PA14_49860	35	49	11.2	4.6E-05	0.61	CCACGCATTATGCTG		
PA14_51660	39	53	11.2	4.7E-05	0.61	CAGTATTATACGCCT		
flhA	82	96	11.3	4.0E-05	0.60	CCGAACAAACTTTTG		
fpvA	27	41	11.3	4.0E-05	0.60	TAAAACGTCATACTA	-1.9	-2.8
glgA	369	383	11.3	4.0E-05	0.60	CAACAGAATGTCCCA		
PA14_03310	233	247	11.3	4.3E-05	0.60	GAGAAGGTTATCCTG		
PA14_22130	251	265	11.3	4.3E-05	0.60	GAAGATTTCATTCTA		
PA14_28920	85	99	11.3	4.2E-05	0.60	CGGGGCAATATGCTG		
PA14_38180	69	83	11.3	4.2E-05	0.60	CAACACAATATTTCC		
PA14_41420	32	46	11.3	4.2E-05	0.60	CGAAACATTATGCGA		
PA14_43430	199	213	11.3	4.3E-05	0.60	TATAATTTTGTTTTA		
PA14_43440	29	43	11.3	4.3E-05	0.60	TAAAACAAAATTATA		
PA14_47420	42	56	11.3	4.3E-05	0.60	CCAGGTAATATACTG		
PA14_47430	12	26	11.3	4.3E-05	0.60	CCAGGTAATATACTG		
rpsA	173	187	11.3	4.2E-05	0.60	CAGGACAATTTGCTG		
aer	339	353	11.4	3.8E-05	0.59	CAGAGGTAAATGCTT		
gcvH2	207	221	11.4	3.8E-05	0.59	CAGAACAAAGTCCCG		
PA14_13140	70	84	11.4	4.0E-05	0.60	ACGAAGATTCTTCTG	-2.5	-2.7
PA14_29575	191	205	11.4	3.8E-05	0.59	TAAAATTTTGCACTT		
PA14_41430	255	269	11.4	3.7E-05	0.59	CAGGACGTCATGCTG		
PA14_59950	124	138	11.4	3.9E-05	0.60	CAGTTGAATCTGCTG		
phzA1	551	565	11.4	3.8E-05	0.59	TAGGATGAAATTTCG		
phzM	131	145	11.4	3.8E-05	0.59	TAGGATGAAATTTCG		
glcB	451	465	11.5	3.6E-05	0.59	CAGGGCTTTATTCTT		
PA14_00520	386	400	11.5	3.6E-05	0.59	TAAAAGATACTATTT		
PA14_30440	293	307	11.5	3.5E-05	0.59	CAACAGTACCTGCTG		
PA14_49650	14	28	11.5	3.5E-05	0.59	ACAAATTAAATGTTG		
PA14_52130	103	117	11.5	3.4E-05	0.59	AAAGGCATTATGCTA		
PA14_54850	353	367	11.5	3.5E-05	0.59	CAACGCTATCTGCTG		
PA14_71190	140	154	11.5	3.6E-05	0.59	CAGAACATCACCCTG		
rbsB	26	40	11.5	3.6E-05	0.59	CAGGATATTTTGCTT		-1.7
cheY	34	48	11.6	3.3E-05	0.58	CAAAAGAATGTGTGG		
edd	116	130	11.6	3.2E-05	0.58	TAGGAGAATCTGCCA		
PA14_26070	90	104	11.6	3.1E-05	0.56	AAATATATTCCGTTG		-3.1
PA14_26080	286	300	11.6	3.1E-05	0.56	CAACGGAATATATTT		-3.0
PA14_48790	4	18	11.6	3.2E-05	0.57	TAAAGTAAAACTTTA		
PA14_54180	352	366	11.6	3.1E-05	0.56	TAATATAACATCCCA		
PA14_63960	109	123	11.6	3.1E-05	0.56	TAGTATATCATTTCT		
PA14_63970	19	33	11.6	3.1E-05	0.56	TAGTATATCATTTCT		
PA14_65210	84	98	11.6	3.1E-05	0.56	AAAATTATTCTGCTG		
putA	98	112	11.6	3.1E-05	0.56	TAATATAACATCCCA		
rnr	107	121	11.6	3.1E-05	0.56	AAAATTATTCTGCTG		
lpxO1	88	102	11.7	2.9E-05	0.56	GAGAATTTACTCCTG		
PA14_54840	266	280	11.7	3.0E-05	0.56	CAGAAGAAAAAATTG		
PA14_54850	65	79	11.7	3.0E-05	0.56	CAGAAGAAAAAATTG		

PA14_58540	28	42	11.7	2.9E-05	0.56	CAGGAGTAAATTCTC		
PA14_59550	325	339	11.7	2.9E-05	0.56	TAAGAGATAATGCCA		
glmM	19	33	11.8	2.7E-05	0.56	GAGCAGAAAATACTT		
PA14_54890	16	30	11.8	2.7E-05	0.56	AAAATTATTGTGCTG		
PA14_68070	27	41	11.8	2.7E-05	0.56	AAGAAGATCCTGCTG	-5.8	-6.0
PA14_71320	53	67	11.8	2.5E-05	0.55	TAAATTATTGTATTA		
alg8	254	268	11.9	2.3E-05	0.53	CAGGATGAAATCCTT		
PA14_05660	124	138	11.9	2.4E-05	0.54	GAGAAGATTCTCCTG		
PA14_08330	59	73	11.9	2.4E-05	0.54	CAGCATCATATTCTT		
PA14_21800	87	101	11.9	2.5E-05	0.54	AAAAATTTTCTGCCG		
PA14_29575	196	210	11.9	2.5E-05	0.54	CAAAATTTTACTTCA		
rdgC	64	78	11.9	2.5E-05	0.54	AAAAATTTTCTGCCG		
orfE	229	243	12	2.1E-05	0.50	TAGCTTTTTATGTTT		
PA14_26730	66	80	12	2.2E-05	0.51	CAGAACAATGAGCTA		
PA14_57680	93	107	12	2.2E-05	0.52	TAGCAGATTACTCCA		
PA14_57690	38	52	12	2.2E-05	0.52	TAGCAGATTACTCCA		
oruR	63	77	12.1	2.0E-05	0.50	CAGAAGATAACCTTG		
PA14_53530	48	62	12.1	2.0E-05	0.50	CAGAAGATAACCTTG		
pcaG	152	166	12.1	2.0E-05	0.50	CAGGAGGATGTGCT		
lpxO2	40	54	12.2	1.7E-05	0.45	TGGAAGATTATGCCG		
mttC	151	165	12.2	1.8E-05	0.45	TAGAACAATCCGTTA		
PA14_27050	82	96	12.2	1.8E-05	0.45	TAGAACAATCCGTTA		
PA14_52140	179	193	12.2	1.7E-05	0.45	TGGAAGATTATGCCG		
nadB	424	438	12.3	1.7E-05	0.45	CAGTACATCGTGTTG		
PA14_23430	63	77	12.3	1.6E-05	0.45	AAATATTATCTGCTT		
PA14_29410	3	17	12.3	1.6E-05	0.45	CAGCAGGTAATGCTT		
PA14_39150	22	36	12.3	1.6E-05	0.45	TAGGACAATCTGTTT		
PA14_39160	112	126	12.3	1.6E-05	0.45	TAGGACAATCTGTTT		
PA14_39670	14	28	12.3	1.7E-05	0.45	AAACACATCATGTTG		
PA14_43440	90	104	12.3	1.6E-05	0.45	GAAAATAAAATCCTT		
PA14_72010	78	92	12.3	1.7E-05	0.45	CAGAACAACACGCTA		
rmd	316	330	12.3	1.7E-05	0.45	CAGAACAACACGCTA		
rplT	63	77	12.3	1.6E-05	0.45	CACAAGAAAATTCTG		
PA14_13940	293	307	12.4	1.5E-05	0.45	CAAGGTTTTATTCTT		
PA14_67120	236	250	12.4	1.5E-05	0.45	CAACGCATTCTGCTG		
amiA	50	64	12.5	1.3E-05	0.44	TAATACGTTATATTA		
PA14_03160	270	284	12.5	1.3E-05	0.44	TAACAGTAAATACTT		-1.5
PA14_03163	291	305	12.5	1.3E-05	0.44	TAACAGTAAATACTT		
PA14_48530	136	150	12.5	1.3E-05	0.44	CAGAAGATTCTGCGG	2.1	2.0
PA14_58500	34	48	12.5	1.3E-05	0.44	TAGAATTTACTGCCA	-2.8	-2.2
PA14_58510	94	108	12.5	1.3E-05	0.44	TAGAATTTACTGCCA	-3.6	-3.4
PA14_73050	36	50	12.5	1.3E-05	0.44	TAATACGTTATATTA		
PA14_28740	35	49	12.6	1.1E-05	0.44	CCGCACTATATACTA		
PA14_30740	427	441	12.6	1.2E-05	0.44	AAAAACATTCTCCTG		
pbpG	234	248	12.6	1.1E-05	0.44	GAGCATTTTATGCTC		
pvdG	66	80	12.6	1.1E-05	0.44	CAACAAATTATGTTG	-4.0	-7.6
pvdS	564	578	12.6	1.1E-05	0.44	CAACAAATTATGTTG		
leuA	166	180	12.7	1.1E-05	0.44	CAATGGAAAATTCTG		
PA14_15050	193	207	12.7	1.1E-05	0.44	CAATGGAAAATTCTG		3.4
PA14_28400	209	223	12.7	1.0E-05	0.44	TCGAACAATGTTCTG		
PA14_28410	142	156	12.7	1.0E-05	0.44	TCGAACAATGTTCTG		

PA14_34290	27	41	12.7	1.1E-05	0.44	TAGGAGAATCTGCTT		
nrdA	302	316	12.8	9.4E-06	0.44	CAAGATAATGCGCTA		
PA14_40790	61	75	12.8	9.8E-06	0.44	TAGAATAAATTTCTA		
PA14_40800	48	62	12.8	9.9E-06	0.44	TAGAAATTTATTCTA		
PA14_49440	417	431	12.8	9.4E-06	0.44	TAGCGCATTATCTTG		
PA14_15850	137	151	12.9	8.6E-06	0.44	CAGAATTTCATGCCA		
PA14_13460	44	58	13.1	6.8E-06	0.37	TATCATTTTATGCTG		
PA14_24960	30	44	13.2	6.3E-06	0.35	AAGAACTATCTGCTG		
PA14_61150	193	207	13.3	5.3E-06	0.31	CAGGAGTTAATTTTA		
PA14_61170	104	118	13.3	5.3E-06	0.31	TAAAATTAACTCCTG		
glgA	84	98	13.4	4.8E-06	0.29	GAAAATAAAATTCTT		
PA14_36560	270	284	13.4	4.8E-06	0.29	GAAAATAAAATTCTT		
PA14_47440	198	212	13.4	4.8E-06	0.29	AAGAACAATATTCTC		
PA14_51590	129	143	13.4	4.8E-06	0.29	TCGAATTATATTCTT		
engB	105	119	13.5	4.6E-06	0.29	AAGTATATAATGCCG		
fliC	212	226	13.5	4.3E-06	0.29	AAAAAGAAAATGTTG		
PA14_60030	28	42	13.5	4.3E-06	0.29	TGGCATATTATGCCA		
PA14_60040	77	91	13.5	4.3E-06	0.29	TGGCATAATATGCCA		
PA14_72470	65	79	13.5	4.6E-06	0.29	AAGTATATAATGCCG		
PA14_73020	79	93	13.5	4.4E-06	0.29	TAAAACGTTATACTA		
PA14_40790	74	88	13.7	3.7E-06	0.29	TAGAAAAATATTCTA		
PA14_40800	35	49	13.7	3.7E-06	0.29	TAGAAAAATATTCTA		
PA14_38180	111	125	14	2.4E-06	0.26	GAAAATAAAATACTA		
PA14_45170	109	123	14	2.3E-06	0.26	CAGAATTTTATGTGA		
PA14_55070	506	520	14	2.5E-06	0.26	AAAGAGATTATTCTG		
PA14_55080	141	155	14	2.5E-06	0.26	AAAGAGATTATTCTG		
PA14_08640	129	143	14.1	2.2E-06	0.26	AAGAACATTCTGCTG		
PA14_34070	12	26	14.2	2.0E-06	0.26	GAAAATTTTATTTTG		
PA14_34080	182	196	14.2	2.0E-06	0.26	GAAAATTTTATTTTG		
PA14_60030	266	280	14.2	1.9E-06	0.26	CAGAATTTTATGTGG		
PA14_43430	194	208	14.3	1.7E-06	0.26	CAAAATTATATTTCG		
PA14_43440	34	48	14.3	1.7E-06	0.26	CAAAATTATATTTCG		
PA14_09570	63	77	14.4	1.5E-06	0.26	TAAAAGAATATTTTT		
PA14_09580	31	45	14.4	1.5E-06	0.26	TAAAAGAATATTTTT		
PA14_62690	168	182	14.5	1.3E-06	0.26	TAAAATAAAATCCTT		3.0
PA14_05500	111	125	14.6	1.1E-06	0.26	CAGAATTTTCTGCTT		
PA14_08640	126	140	14.7	9.5E-07	0.26	CAGAAGAACATTCTG		
PA14_68110	138	152	14.7	9.8E-07	0.26	TAAAACAATATCTTG		
PA14_26420	272	286	15	7.2E-07	0.26	TAAAATGTTATACTA		
PA14_26450	351	365	15	7.2E-07	0.26	TAAAATGTTATACTA		

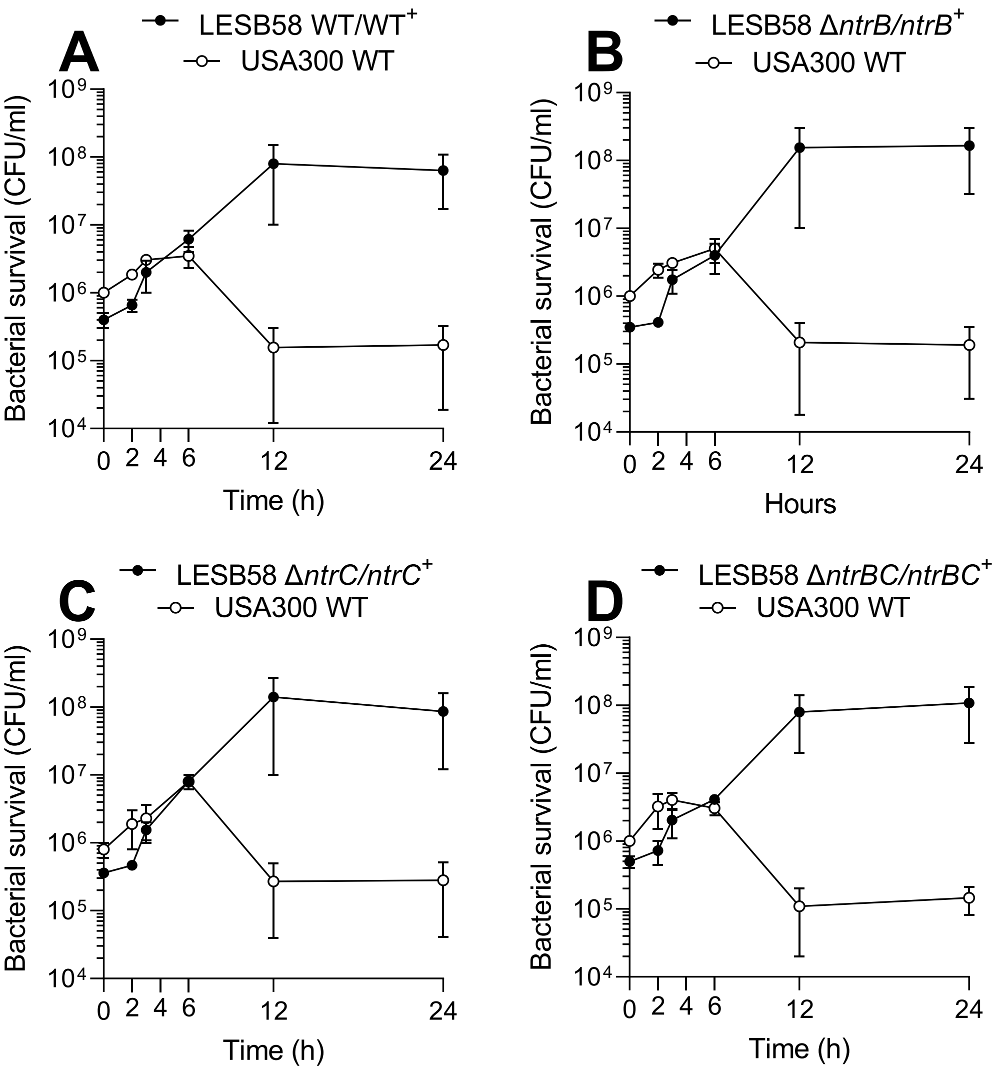

Figure S1. Complementation of *P. aeruginosa* LESB58 Δ*ntrB*, Δ*ntrC* and Δ*ntrBC* strains restored interspecies competition with *S. aureus* USA300. *P. aeruginosa* LESB58 **(A)** WT, **(B)** Δ*ntrB/ntrB*$^+$, **(C)** Δ*ntrC/ntrC*$^+$ or **(D)** Δ*ntrBC/ntrBC*$^+$ complemented strains were seeded at a starting OD_{600} = 0.1 in batch cultures that were sampled in 2-, 6- or 12-h intervals and plated on selective media for bacterial enumeration. Data are presented as mean ± standard error of the mean (SEM) for three independent experiments (*n* = 3).

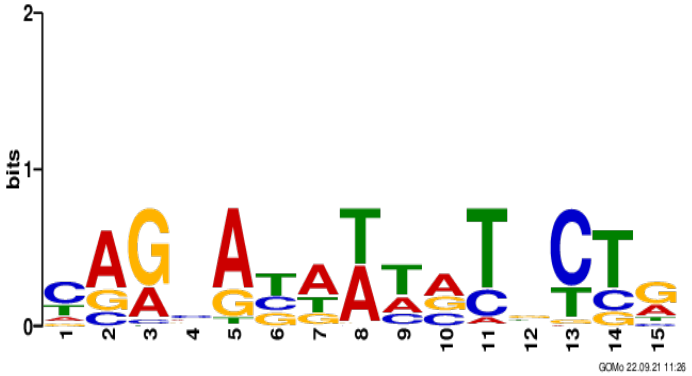

Figure S2. NtrC binding motif calculated using Autoseed software followed by manual refinement with available HT-SELEX and ChIP-Seq data.
